# Towards AI-Driven Healthcare: Systematic Optimization, Linguistic Analysis, and Clinicians’ Evaluation of Large Language Models for Smoking Cessation Interventions

**DOI:** 10.1145/3613904.3641965

**Published:** 2024-05-11

**Authors:** Paul Calle, Ruosi Shao, Yunlong Liu, Emily T. Hébert, Darla Kendzor, Jordan Neil, Michael Businelle, Chongle Pan

**Affiliations:** School of Computer Science, University of Oklahoma Norman, Oklahoma, USA; TSET Health Promotion Research Center, Stephenson Cancer Center, University of Oklahoma Health Sciences Center Oklahoma City, Oklahoma, USA; School of Computer Science, University of Oklahoma Norman, Oklahoma, USA; School of Public Health, The University of Texas Health Science Center at Houston Austin, TX, USA; TSET Health Promotion Research Center, Stephenson Cancer Center; Department of Family and Preventive Medicine, University of Oklahoma Health Sciences Center Oklahoma City, Oklahoma, USA; TSET Health Promotion Research Center, Stephenson Cancer Center; Department of Family and Preventive Medicine, University of Oklahoma Health Sciences Center Oklahoma City, Oklahoma, USA; TSET Health Promotion Research Center, Stephenson Cancer Center; Department of Family and Preventive Medicine, University of Oklahoma Health Sciences Center Oklahoma City, Oklahoma, USA; School of Computer Science, University of Oklahoma Norman, Oklahoma, USA

**Keywords:** Large Language Model, Message Generation, Computational Linguistic Analysis, Expert Review, Smoking Cessation Intervention

## Abstract

Creating intervention messages for smoking cessation is a labor-intensive process. Advances in Large Language Models (LLMs) offer a promising alternative for automated message generation. Two critical questions remain: 1) How to optimize LLMs to mimic human expert writing, and 2) Do LLM-generated messages meet clinical standards? We systematically examined the message generation and evaluation processes through three studies investigating prompt engineering (Study 1), decoding optimization (Study 2), and expert review (Study 3). We employed computational linguistic analysis in LLM assessment and established a comprehensive evaluation framework, incorporating automated metrics, linguistic attributes, and expert evaluations. Certified tobacco treatment specialists assessed the quality, accuracy, credibility, and persuasiveness of LLM-generated messages, using expert-written messages as the benchmark. Results indicate that larger LLMs, including ChatGPT, OPT-13B, and OPT-30B, can effectively emulate expert writing to generate well-written, accurate, and persuasive messages, thereby demonstrating the capability of LLMs in augmenting clinical practices of smoking cessation interventions.

## 1 INTRODUCTION

Smartphone-delivered message intervention has become an essential part of an effective smoking cessation program. Meta-analytical reviews have demonstrated the efficacy of receiving smartphone-delivered message interventions via short message service (SMS) or smartphone applications in promoting both short-term self-reported quitting behaviors and long-term abstinence [82, 85, 93]. Compared to traditional face-to-face counseling interventions, a short intervention message can offer real-time and tailored support when individuals are vulnerable to lapse or relapse of smoking, thereby significantly enhancing tobacco abstinence rates [21, 43, 70, 97]. Research in the domain of smoking cessation treatment and behavioral intervention broadly calls for the integration of smartphone-delivered message interventions to improve treatment efficacy [31, 57, 98].

Despite the well-established efficacy of smartphone-delivered message interventions, maintaining user engagement and intervention effectiveness rely on the provision of novel and non-repetitive content [69]. Repetition in messages can elicit perceptions of redundancy and a feeling of information overload, which further diminish the perceived utility of the received information and attenuate attitudinal and behavioral changes [86]. Qualitative feedback echoes this observation; for example, in [14] participants who received a text messaging intervention designed to provide nutrition education and encourage better dietary choices suggested that future designs of text messaging intervention should avoid content repetition and offer an opportunity to learn new information for each interaction message [14]. Consequently, the development of effective smoking cessation treatment necessitates the creation of substantial, high-quality intervention messages, a labor-intensive process for experts that poses a significant challenge.

In the field of Natural Language Processing (NLP), the advent of Large Language Models (LLMs) has significantly facilitated the capability of message generation. These LLMs, equipped with vast training datasets, can not only mimic human writing but also draw upon the knowledge embedded in the training data to produce fluent text [12, 15]. Previous research has examined the feasibility of LLMs for message generation in diverse health-related contexts. For example, Karinshak et al. [41] compared COVID-19 pro-vaccination messages generated by the GPT-3 model with human-authored messages released by the Centers for Disease Control and Prevention (CDC). Their findings revealed that messages produced by GPT-3 were perceived by crowdworkers as more effective, presenting stronger arguments, and evoking more positive attitudes about vaccination. Similarly, Lim and Schmalzle [50] undertook a comparison of health awareness messages on folic acid generated by the BLOOM-7B1 model with tweets on the same topic. Their study demonstrated that LLM-generated messages were rated higher in terms of message quality and clarity, compared to human-written tweets. Furthermore, computational text analysis indicated that LLM-generated messages exhibited similar characteristics to those written by humans in terms of sentiment, reading ease, and semantic content.

While prior research has supported the feasibility of LLMs in message generation [15, 41, 50], using LLMs to generate intervention messages for smoking cessation treatment necessitates the exploration of two critical questions: 1) How can LLMs be optimized to mimic human expert writing, and 2) Do LLM-generated messages meet clinical standards to be safely implemented in tobacco treatment programs? To address these pivotal questions, the present study conducted a systematic examination of the message generation and evaluation processes of LLMs within the context of smoking cessation intervention.

**Figure 1:**
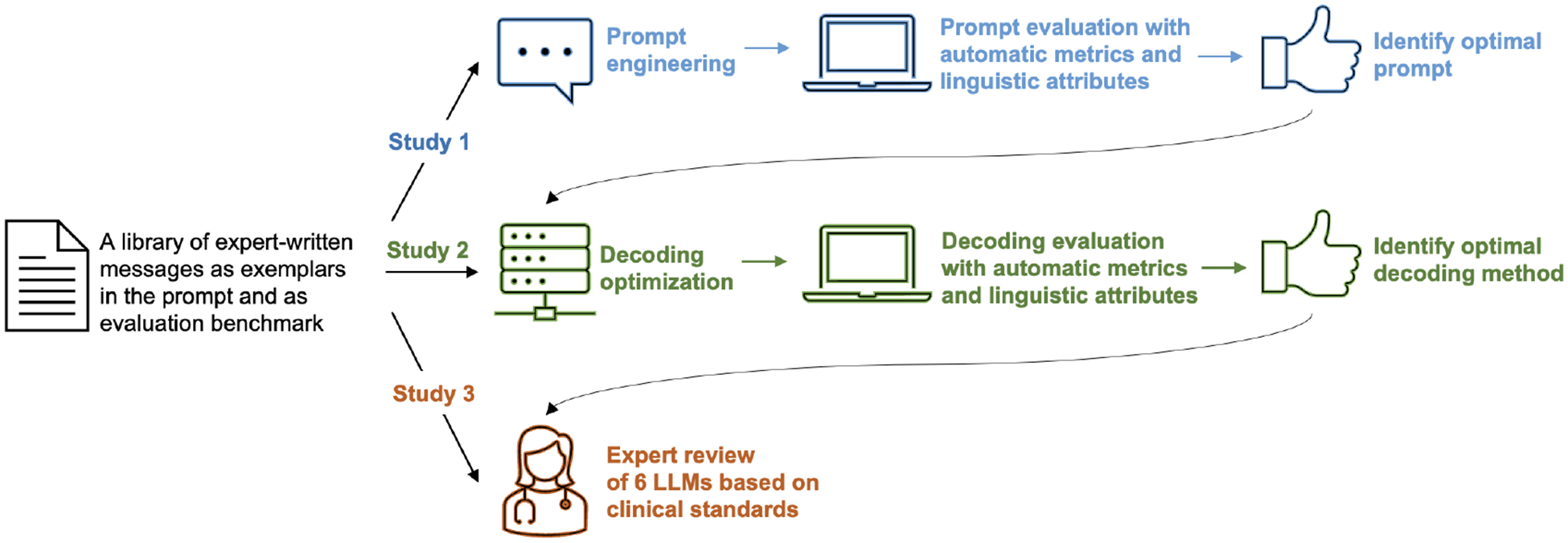
An overview of the three studies, including prompt engineering (Study 1), decoding optimization (Study 2) and expert review (Study 3).

In the message generation process of LLMs, both the prompt choice and the decoding method serve as critical determinants that influence the quality of the generated text [3]. To refine LLMs to emulate human expert writing, this study conducted prompt engineering (Study 1) and decoding optimization (Study 2), involving the testing of five prompts and eight decoding methods to generate intervention messages across five LLMs (see Figure 1). The evaluation of LLM performance in message generation is a multi-faceted task, requiring a comprehensive assessment of message quality, coherence, relevance, grammaticality, and accuracy. Consequently, the employment of commonly adopted automatic metrics in LLM assessment is often oversimplified and inadequate in capturing the nuanced linguistic properties and overall text quality [4, 9, 75, 81]. In recognition of these challenges, our study introduced a comprehensive evaluation framework encompassing diversity, quality, and efficiency. We built upon the computational linguistic approach and utilized the Linguistic Inquiry and Word Count (LIWC) tool to analyze linguistic features of LLM-generated messages [87], using intervention messages written by tobacco treatment experts as the benchmark. In Studies 1 and 2, we identified the prompt and decoding methods that most closely resembled human expert writing. These strategies were subsequently recommended and subjected to evaluation in terms of their clinical utility (Study 3).

While LLMs have demonstrated their capacity in generating effective public health messages [41, 50] and have exhibited knowledge levels comparable to third-year medical students [29, 44, 83], their applicability in the high-stakes healthcare context necessitates rigorous evaluations to ensure both reliability and safety. Previous research underscores the importance of expert review in increasing the validity of LLM evaluations, especially for tasks requiring specialized expertise [9, 23, 38, 48, 56, 95]. Therefore, to establish the efficacy of LLMs in generating intervention messages for clinical use, we invited certified tobacco treatment specialists (TTS) to conduct an expert review (Study 3). These specialists rigorously evaluated the messages generated by the five LLMs from Studies 1 and 2, and the newly released ChatGPT, on message quality, accuracy, credibility, and persuasiveness, using expert-written messages as the benchmark. Drawing upon their extensive clinical experiences in tobacco treatment counseling, they further evaluated whether the generated messages met the standards in TTS training and were ready to use in clinical practice. Our results indicate that larger LLMs, including ChatGPT, OPT-13B and OPT-30B, can effectively emulate human expert writing to generate well-written, accurate, credible, and persuasive messages, thereby demonstrating the efficacy of LLMs in augmenting smoking cessation interventions in clinical settings.

Our paper contributes to the current research landscape on several key aspects. First, we conducted a systematic examination of message generation and evaluation with LLMs, involving prompt engineering, decoding optimization, and expert review, conducted across six state-of-art LLMs, thereby ensuring the generalizability of our findings. Furthermore, we proposed an innovative and comprehensive evaluation framework for LLM assessment, integrating automatic metrics, linguistic attributes, and expert evaluation, offering a multifaceted assessment that delves into diversity, quality, and efficiency aspects of the LLM performance. To date, this is the first study that adopted the computational linguistic approach for the assessment of LLMs and established the efficacy of LLMs in specialized healthcare contexts through expert reviews.

This paper is organized as follows: Section II and III address the first research question on prompt engineering and decoding optimization. Section II provides an overview of prompt engineering literature, introduces the LLMs employed for message generation, presents a multi-faceted evaluation framework, and assesses prompts accordingly. Section III reviews literature on decoding parameters and examines how decoding methods influence message generation. Section IV addresses the second research question by incorporating expert reviews of LLM-generated intervention messages. Section V discusses future applications and limitations of LLMs in the healthcare context, and Section VI concludes.

## 2 STUDY I: PROMPT ENGINEERING

### 2.1 Related Work

Large language models (LLMs) are deep learning models trained to understand and generate natural language. Autoregressive LLMs work by using a series of input tokens (words or fragments thereof) to generate subsequent tokens [15, 73]. Grounded in the transformer architecture, which is a cutting-edge neural network design characterized by its massive parameter sizes, LLMs can successfully understand patterns within the input tokens through a self-attention mechanism [89]. In recent years, the efficacy of LLMs has been examined and validated for various natural language tasks, including automatic summarization, machine translation, and question answering [15, 60].

#### 2.1.1 Large Language Models.

Currently, there are open-source and closed-source models based on the availability of model weights. Leading open-source models in the field include GPT-J-6B, the BLOOM series, and the OPT series. For the purposes of this research, we employed models compatible with our computational resources, namely GPT-J-6B [90], Bloom-7B1 [78], and OPT 6.7B, 13B, and 30B [100]. GPT-J-6B is an open-access alternative to GPT-3 and was trained using the Pile dataset [11], a predominantly English-centric text corpus that combines sources such as English Wikipedia and PubMed Central. BLOOM introduces modifications to the conventional Transformer architecture [89], including the incorporation of ALiBi Positional Embeddings [72] and the Embedding LayerNormal. BLOOM is trained with the ROOTS dataset [46], a multi-lingual text corpus. The architecture of OPT is similar to GPT-3 and was trained on a corpus comprised by subdatasets of RoBERTa [53], a subset of the Pile, and a subset of the Pushshift dataset [8]. The primary language of OPT’s corpus is English. We used LLMs of diverse sizes, ranging from 6 billion parameters (as in GPT-J-6B) to 30 billion parameters (as in OPT-30B) to identify the optimal model for message generation in the healthcare context and to enhance the generalizability of our findings.

#### 2.1.2 Prompt engineering for LLMs.

We employed the “tuning-free prompting” approach [25, 52, 92, 102] to generate intervention messages, a method where the parameters/weights of the model are fixed and in-context learning is used [15]. This approach leverages the input context to guide the model’s responses without the need for task-specific fine-tuning. For example, to instruct LLMs in generating intervention messages for smoking cessation, we can provide a handful of intervention messages as examples to help models infer the format of message output. The number of examples provided can be used to differentiate the learning process into three categories: zero-shot, one-shot, and few-shot. In zero-shot learning, the model is given a natural language description of the task without any examples, whereas one-shot and few-shot learning provide the model with one or a few context-relevant examples. As concluded by Brown et al. [15], LLMs are meta-learners that integrate outer-loop gradient descent learning with in-context learning, and few-shot learning, by prepending context-specific examples, can greatly enhance model performance and adaptation to task contexts, especially in areas requiring specialized knowledge and messaging styles, such as smoking cessation intervention.

For message generation tasks using few-shot learning, a prompt is defined as the initial input provided to the LLM for generating message output [52]. In our study, this input comprises both the task instruction and the message exemplars authored by human experts. For example, when prompting the BLOOM-7B1 model with this task instruction “Write motivational messages to encourage people to quit smoking:” along with four sample messages written by human experts, the model returned the following message:

“If you know that you are having a strong craving, you can take a deep breath and relax your muscles. When you can relax, you can focus on something else, like reading or watching TV. Do not attempt to fight the urge to smoke. Instead, take the next deep breath and wait for it to pass.”

Both the prompt and the accompanying examples can influence the quality and relevance of the model output. Prompting serves multiple purposes: setting the context, clarifying the nature of the task, guiding response format, improving relevance and accuracy, and reducing ambiguity and potential biases [15, 53, 73]. As high-lighted by the PromptBench benchmark [103], LLMs are sensitive to prompts, underscoring the importance of prompt engineering for optimal model performance. Therefore, enhancing model efficacy necessitates prompt engineering, an iterative refinement process which involves rephasing the prompt for clarity and precision, providing context-relevant examples, and specifying response format.

In this study, we employed manual template engineering to generate prompts[52], (see Table 1). The first three prompts were adapted from [15], with each subsequent prompt version increased in length and detail. The first version provided a broad instruction with no context (“Message:”), the second version provided a specific theme for the message output (“Messages to help you quit smoking:”), and the third version instructed explicitly on both message theme and desired tone (“Write motivational messages to encourage people to quit smoking:”). Further, the fourth version introduced a variation in placement by positioning the task instruction after the example messages (Example messages + “Write messages like the previous ones:”). By prepending message exemplars before the general instruction ‘Write messages like the previous ones,’ we were able to test whether providing context-related exemplars and having LLMs infer the task instructions, without explicitly conditioning the tasks, themes, or tones, would lead to more relevant and accurate output. Lastly, the fifth version adopted a structured format [51, 79], using tags to label the task and examples (“Task: Write messages that are on the same topic” + Message 1: … + Message 2: …). Structured prompting was introduced to help LLMs better distinguish between task instructions and exemplars and its effectiveness in increasing the accuracy, relevance, and context appropriateness of the model’s output was further examined. The message example dataset contains intervention messages from existing research on smoking cessation intervention written by tobacco treatment experts [16, 17, 35, 36].

#### 2.1.3 Perplexity and Computational Linguistic Analysis.

This study first evaluated prompt performance with the perplexity measure [6]. Perplexity is a standard metric for evaluating the quality of language models and quantifies how “surprised” the model is when seeing a passage of text [24]. A passage of text with a perplexity too high may contain language errors or nonsensical content [30], while a perplexity too low may signify repetitive and uninteresting text [34].

The words we use in communication not only reflect our identity but also the social and relational context in which communication takes place. Linguistic features, such as emotional tones or the use of complex words, allow individuals to infer the medical expertise of their healthcare provider, subsequently influencing their trust and adherence to health-related messages [88]. To explore the extent to which LLMs emulate human expert writing, we examined the psychometric properties and linguistic features in LLM-generated messages, using human expert writing as the benchmark. We adopted the computational linguistic approach, which involves quantifying linguistic patterns and psychometric properties in a given text by investigating word usage within predefined psychometric categories [87]. In this study, five critical linguistic features, including word count, clout, emotional tone, authenticity, and use of complex words, were chosen to evaluate the properties of the intervention messages [13]. The clout score is a proxy for relative social status, confidence, or leadership. A high clout score indicates greater expertise, certainty, and confidence in communication, whereas a low score suggests a tentative style of expression[40, 59]. Authenticity reflects the varying degrees of personal and disclosing styles in discourse [63]. Higher authenticity score indicates honest, personal, and disclosing communication, whereas lower score implies a distanced and impersonal style. Emotional tone ranges from negative (values <50) to positive (values >50), with a midpoint of 50 on the 100-point scale representing a neutral emotional tone [26]. Scores for clout, authenticity, and emotional tone are standardized composite variables transformed to a scale ranging from 1 to 100. Complex words refers to the proportion of words that are 7 letters or longer [13].

The five selected linguistic features signify the quality, credibility, and trustworthiness of writings in the context of substance use intervention and health communication. Partch and Dykeman [68] analyzed the linguistic attributes of text messages used in substance use disorder treatments and found that, in comparison to everyday Twitter posts, intervention messages exhibited greater clout, maintained neutral emotional tones, and displayed lower authenticity. Additionally, Toma & D’Angelo [88] posited that medical advice messages with a higher word count and frequent utilization of complex words were perceived as more authoritative. Together, previous literature has identified linguistic markers that bolster the perception of credibility and trustworthiness in health intervention messages: a higher word count and greater clout reduce uncertainties and provide comprehensive and assertive explanations; neutral emotional tones and impersonal writing foster a psychologically detached and objective stance; and the employment of complex words signifies cognitive complexity and thereby enhances the perceptions of formality, expertise, and professionalism.

### 2.2 Method

A library of 899 intervention messages written by tobacco treatment experts and validated in clinical trials served as the training and validation data [16, 17, 35]. Drawing upon research in few-shot learning, which has demonstrated significant improvements in text generation performance when increasing the number of examples from 1 to 4, with diminishing returns beyond 4 examples [101], we opted to use 4 message exemplars in combination with each of the 5 prompt versions under examination (refer to Table 1 for 5 prompt versions and refer to Table 2 for an example of the prompt input with message exemplars). These exemplars were randomly selected from the human-written message library and used as input for 5 state-or-art LLMs, namely GPT-J-6B, Bloom 7B1, and OPT 6.7B, 13B, and 30B. The message generation process was repeated 100 times for each LLM, utilizing the Transformer library from HuggingFace. Computational tasks were performed on a server with an AMD Ryzen Threadripper PRO 3955Wx (16-Cores, 32-Threads) and two Nvidia RTX A6000 48GB GPUs with the Ubuntu 20.04.4 LTS OS. The initial process yielded a total of 21,638 messages.

**Figure 2:**
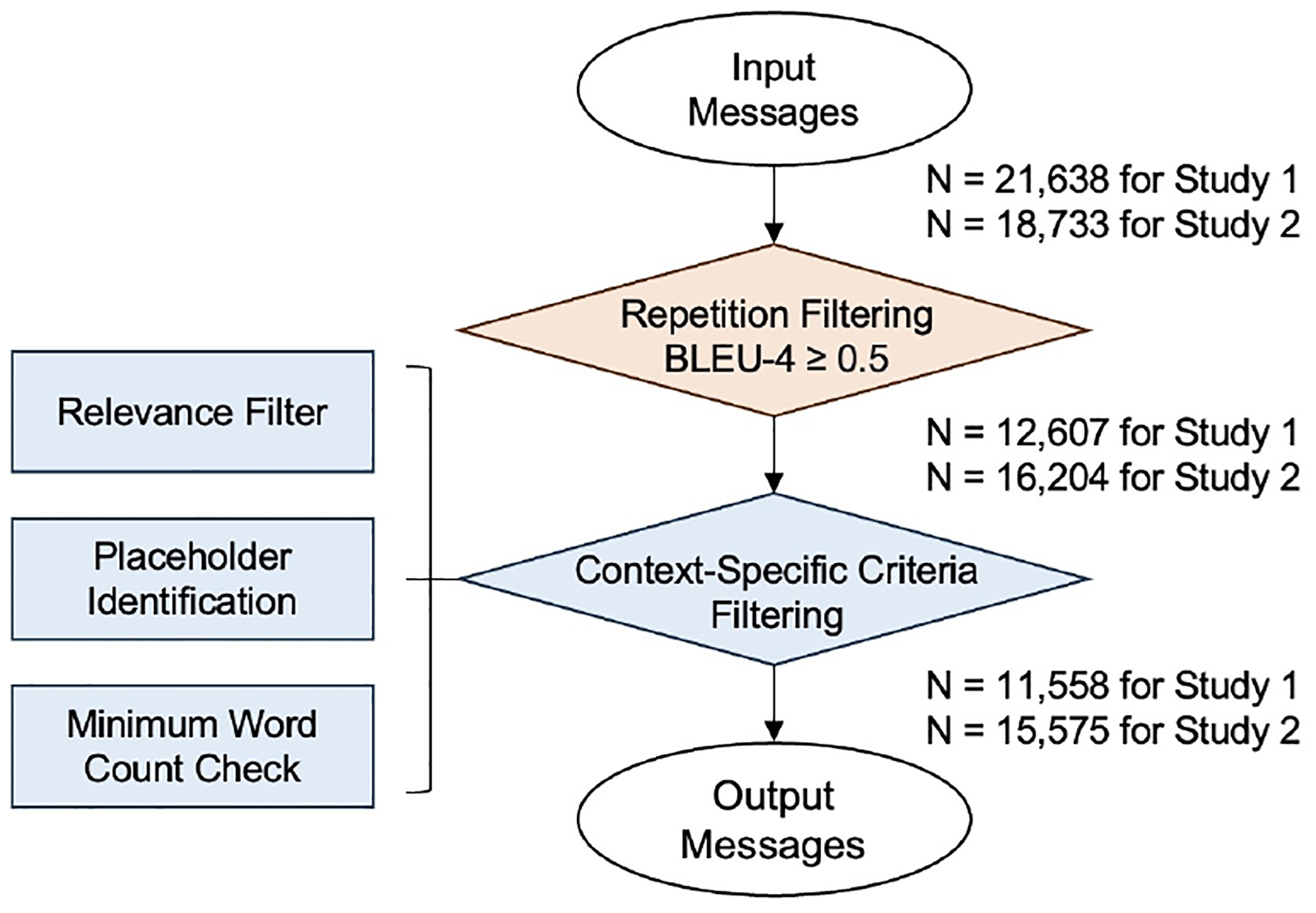
Two-step message filtering with sample sizes at each step for studies 1 and 2

To ensure relevancy of LLM-generated messages, this study employed a two-step filtering process (see Figure 2). In the first step, BLEU-4 scores were computed to measure repetition between each message and the rest of the generated messages from the same LLM [67, 104]. The BLEU-4 metric evaluates the similarity up to 4 consecutive words between pairs of intervention messages, producing scores that range from 0 to 1, with values nearing 1 indicating high similarity between messages. We set the threshold at 0.5 to discard redundant messages with BLEU-4 scores equal to or larger than 0.5. In the second step, messages were filtered out based on three criteria tailored to the context of smoking cessation and the characteristics of the example dataset: (1) the presence of words like “app”, “apps”, or “applications”. This criterion was applied because the example data contained messages designed for smartphone-based smoking cessation interventions, and a small portion of which aimed to promote app engagement rather than smoking cessation itself. (2) the inclusion of underscores (“_”), which indicated placeholders for information to be inserted. And (3) messages consisting of fewer than six words. This threshold was chosen because expert-written messages contain at least six words, and messages shorter than six words tend to lack the necessary informativeness for effective intervention. Subsequent message filtering based on repetition (BLEU-4 ≥ 0.5) and three context-specific criteria resulted in a final count of 11,558 messages. We combined the filtered LLM-generated messages (N=11,558) with the original human-written messages (N=899) to create the final sample for evaluation (N=12,457).

Five prompts were rigorously assessed based on three key dimensions: diversity, quality, and efficiency of the generated messages. *Diversity* was evaluated using two primary indicators: perplexity scores and the pass rate of repetition filtering. Perplexity scores for each prompt version and each LLM were computed, utilizing the perplexity score from the expert-writing condition as the gold standard. The selection of the optimal prompt was predicated on its performance in close proximity to this reference perplexity. Importantly, this study refrained from the pursuit of lower perplexity scores, as such metrics could potentially signify a lack of diversity in the generated messages [34, 67]. *Quality* was ascertained through a comprehensive analysis of critical linguistic features, using expert-written messages as the benchmark. Linguistic attributes, including word count, clout, emotional tone, authenticity, and the use of complex words, were quantified for each generated message using the Linguistic Inquiry and Word Count (LIWC) software (LIWC-22, version 1.3.0) [13]. Efficiency was gauged by calculating the average number of messages retained after both repetition and criteria-based filtering applied to each iteration. This measure served as a practical indicator of the prompt’s efficiency in generating messages that not only meet quality standards but also do so in an efficient manner.

### 2.3 Results

In the initial message generation phase, each of the five prompt versions yielded an average of 6.7 to 13.8 messages per iteration. The initial repetition filtering step discarded between 23.5% and 72.8% of these messages. Subsequent criteria-based filtering removed an additional 3.2% to 7.0% of these messages. As a result, the proportion of messages retained, relative to the initial count generated by each prompt version, ranged from 20.2% to 73.3%. On average, the human-authored messages comprised 26.16 words (SD=10.80). They exhibited high clout (M=76.62, SD=29.90), a low degree of authenticity (M=43.74, SD=37.24), and a neutral emotional tone (M=48.79, SD=38.97). These messages contained approximately 22% complex words (SD=10.74).

**Figure 3:**
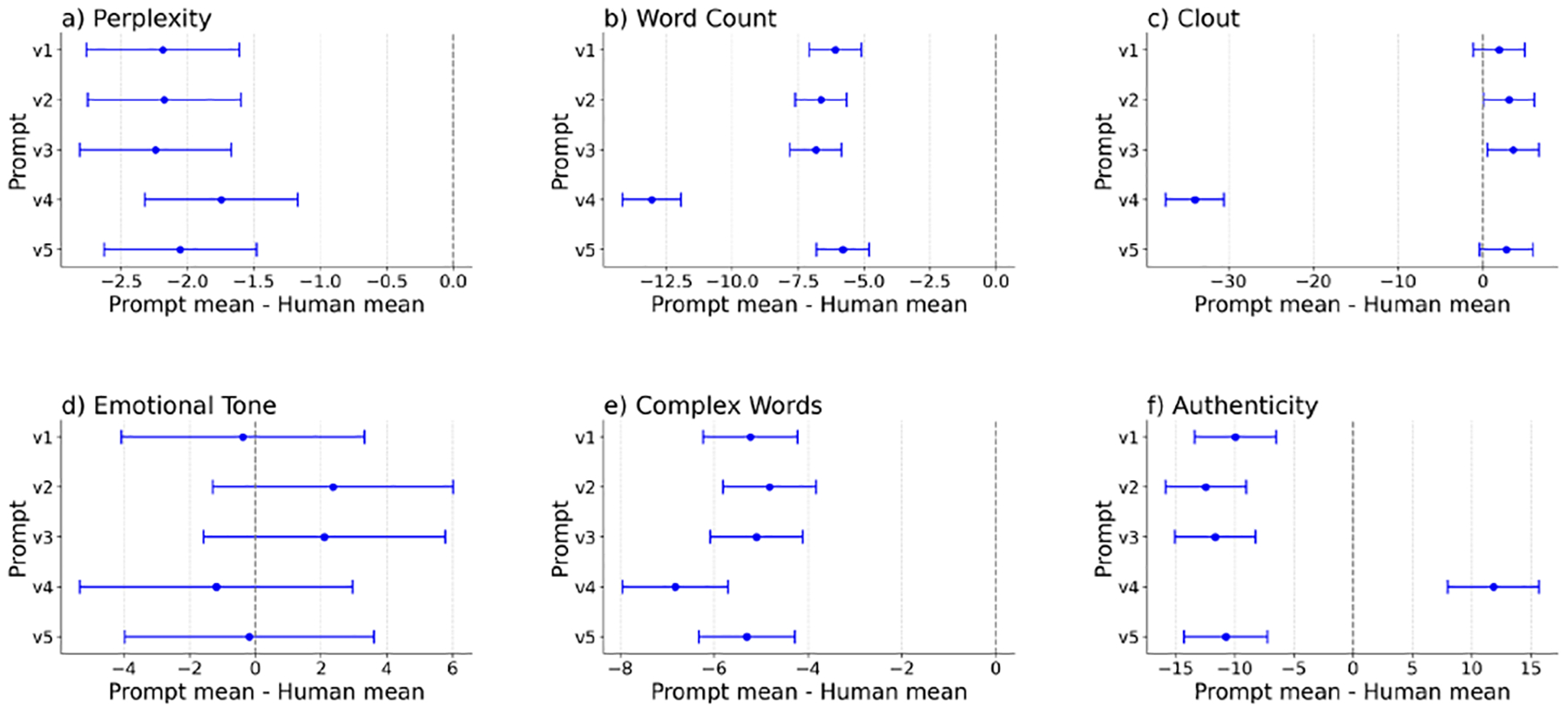
Confidence intervals for Dunnett test for prompt mean - human mean

Five one-way analysis of variance (ANOVA) tests with post-hoc multiple comparison analyses were conducted to compare linguistic features of messages generated with the five different prompts with expert generated messages. Results indicated that messages generated with the prompts significantly differed from human-written content in terms of word count (F(5, 12451) = 172.42, p < .001, partial *η*^2^ = .07), clout (F(5, 12451) = 293.29, p < .001, partial *η*^2^ = .11), authenticity (F(5, 12451) = 102.95, p < .001, partial *η*^2^ = .04), emotional tone (F(5, 12451) = 2.97, p = .011, partial *η*^2^ = .00), and the use of complex words (F(5, 12451) = 48.20, p < .001, partial *η*^2^ = .02). Subsequent comparisons using Dunnett’s test were conducted between each prompt and the expert-writing condition (Figure 3). Results demonstrated that compared with human-written messages, messages generated with five different prompts consistently had fewer words and used less complex vocabulary (all p values <.001). Compared with expert generated messages, messages generated with prompt version 4 exhibited higher authenticity scores, whereas messages generated with the other four prompts had lower authenticity scores (all p values <.001). In addition, both the generated messages and the expert generated messages used a relatively neutral emotional tone (all p values >.05). Messages generated with prompt versions 1 and 5 displayed clout similar to human writing, whereas messages generated with prompt versions 2 and 3 had significantly higher clout and those generated with prompt version 4 had significantly lower clout. The ANOVA test with post-hoc multiple comparison analysis revealed significant differences in perplexity between each prompt with the expert-writing condition (F(5, 99) = 20.87, p < .001, partial *η*^2^ = .51). Post-hoc Dunnett’s test further indicated that LLMs using all five prompts had lower perplexity (M ~ 4.29 to 4.79; SD ~ .39 to .66) compared to messages written by human experts (M=6.54, SD=.63, all p values <.001).

### 2.4 Discussion

Based on a comprehensive evaluation encompassing diversity, quality, and efficiency, our findings highlight the significant impact of different prompts on the ability of LLMs to generate intervention messages, assessed against the expert-written benchmark. Despite advances in LLMs, a discernible gap remains between machine-generated and human-written messages. Across all five prompt versions, LLM-generated messages exhibited significant deviations from the expert-writing condition in terms of word count, authenticity, the use of complex vocabulary, and model perplexity. Specifically, messages generated by LLMs were less detailed (evidenced by a reduced word count), more formal and monotonous (with lower authenticity, except for those generated with prompt version 4), less professional (utilized less complex vocabulary), and less diverse (indicated by lower perplexity scores). Notably, our findings indicate that the sequencing of task instructions after message examples, as observed in prompt version 4, adversely impacted the performance of all evaluated Large Language Models (LLMs), extending the ordering effects of training examples on model performance to the relative placement of instructions and examples within prompts [101]. In particular, the “example + instruction” arrangement led to highly repetitive outputs, with a repetition rate of 72.8% compared to rates ranging from 23.5% to 33% for other prompts. Additionally, the quality of messages generated using this prompt was suboptimal, as evidenced by a 7% message discard rate due to criteria-based filtering, compared to discard rates of 3.2% to 3.8% for other prompts. Furthermore, linguistic features of these messages diverged significantly from human writing across all critical dimensions. Finally, the production efficiency of messages generated using this prompt was markedly lower, with a pass rate of 20%, in contrast to pass rates ranging from 62.8% to 73.3% for other prompts.

Prompt versions 1, 2, and 3 in our study featured task-specific instructions that ranged from a general directive (“Message:”) to more detailed guidelines with a constrained tone (“motivational”) and theme (“smoking cessation”). Existing literature posits that detailed and specific prompts act as semantically meaningful task instructions, thereby facilitating more efficient model learning — akin to how specific task instructions enhance human learning efficiency [15, 61, 79]. This aligns with a common assumption in few-shot learning research, which suggests that optimal model performance necessitates expertly crafted, clear, and accurate task descriptions [54, 79]. Contrary to these expectations, our findings indicated that the general instruction (prompt version 1) outperformed its more detailed and specific counterparts (prompt versions 2 and 3) across metrics of message diversity, quality, and efficiency. Specifically, messages generated using prompt version 1 exhibited performance closely aligned with human expert-generated messages in terms of perplexity and key linguistic features such as clout and emotional tone. Moreover, after applying a two-step filtering process, prompt version 1 yielded an average of 7.1 messages per iteration, achieving higher efficiency for message generation compared to 6.6 messages for prompt version 2 and 6.8 messages for prompt version 3.

The finding that a general prompt outperformed detailed and specific ones, although counterintuitive, in fact echoes emerging research that questions the necessity for models to receive semantically meaningful instructions [61, 71, 91]. Empirical studies have shown that, in a few-shot learning context, models perform comparably well when given irrelevant or misleading instructions as opposed to clear and specific directives. This suggests that prompts may serve more to help models learn the distribution of input text rather than to provide explicit task instructions [45, 60, 91]. Furthermore, our findings align with research by Yang et al [97], which demonstrated that prompts with a structured format (referred as schema-based prompts, e.g., “Title: ....; Author....”) consistently outperform natural language (NL)-based prompts (referred as template prompts, e.g., “The title is ....; The author is ....”) in few-shot learning across various NLP tasks. In our study, both prompt version 1 (“Messages:”) and version 5 (“Task: Write messages that are on the same topic” + Message1: …+ Message2: …) adopted a structured format and outperformed the natural language sentences employed for task-specific instructions in prompt versions 2 and 3. Despite comparable quality in the messages generated by prompt versions 1 and 5, the more succinct version 1 exhibited higher efficiency, yielding an average of 7.1 messages per iteration, as compared to an average of 3.5 messages generated by prompt version 5. These findings suggest a general pattern within the context of few-shot learning: model performance benefits from both the brevity and the structured nature of the prompt.

## 3 STUDY II: DECODING OPTIMIZATION

### 3.1 Related Work

A decoding method interprets LLM’s output probabilities for the subsequent token (word, sub-word, or character) in a sequence and select the most suitable one for text generation [1]. Various decoding methods exist, each with its own set of parameters. The selection of decoding methods and parameter values needs to balance the quality and diversity of the message output. In this study, we employed three decoding methods, namely temperature sampling, top-k sampling, and nucleus sampling, due to their extensive application in text generation tasks [1, 16, 36].

Temperature sampling modulates the softmax output probabilities using a temperature parameter T. As the temperature value approaches 0, the distribution becomes peakier, thereby favoring the most probable tokens and leading to more deterministic but potentially less diverse output. Conversely, a temperature close to 1 keep the same distribution, increasing the likelihood of sampling less probable tokens. Hashimoto et al. [32] explored the balance between diversity and quality in temperature annealing across single-sentence natural language generation tasks, including summarization, story generation, and chit-chat dialogue. Their findings revealed a quality-diversity trade-off: while lowering the temperature (T=0.7, as compared to T=1) improved the quality of generated text, it also reduced diversity and introduced repetition issues across all three tasks. Similarly, Holtzman et al [34] observed that temperatures exceeding 0.9 yielded messages diversity akin to human writing, measured by self-BLEU scores, and temperatures above 0.7 effectively mitigated repetition issues. In the healthcare context, Schmalzle and Wilcox [80] conducted a pilot test employing temperature settings of 0.3, 0.5, 0.7, and 1 with a fine-tuned 355M GPT-2 model, aiming to create messages about folic acid. Their results indicated that T=0.7 produced the most balanced outputs in terms of both quality and diversity, whereas T=1 led to incoherent outputs and T<0.5 produced text that was highly homogeneous with the training text. In light of these observations, previous studies generally recommend an optimal temperature threshold within the range of [0.7, 1] for text generation tasks.

Temperature sampling is frequently used in conjunction with other techniques such as top-k or top-p sampling. In top-k sampling, the k most probable subsequent tokens are filtered, and the probability mass is then redistributed exclusively among these k options. This approach introduces a degree of stochasticity into the decoding process, thereby enriching the diversity of the generated text relative to other methods like greedy decoding or beam search. Previous research commonly recommended combining top-k sampling with a temperature setting of T=0.7, as in [28, 73]. The optimal value of k may vary depending on the specific task and requirements [37]. For example, Fan et al. [28] employed k=2 for summarization tasks and k=40 for text generation. Similarly, in [37], k=10 was recommended for story generation, aiming to produce text that is coherent, contextually relevant, and diverse. Nucleus sampling, also known as top-p sampling, deviates from the fixed-number approach inherent in top-k sampling. Instead, it selects tokens from the smallest subset that has a cumulative probability exceeding a predefined threshold p [34]. This method allows for dynamic adjustments in the number of tokens considered at each decoding step, thereby enhancing the diversity and creativity of the generated text. Holtzman et al. [34] observed that nucleus sampling with p=0.95 closely matched human performance in terms of both perplexity and self-BLEU scores. Additionally, De Lucia et al. [27] recommended an optimal p-value range of [0.7, 0.9] for narrative generation using GPT-2, based on four evaluative criteria: interestingness, coherence, fluency, and relevance.

In message generation tasks, the selection of decoding methods is critical for achieving the optimal balance between diversity and quality [18, 28, 32, 33, 37]. A systematic comparison of temperature sampling, top-k sampling, and nucleus sampling has indicated that these sampling strategies can yield comparable performance with tuned hyperparameters [62, 99]. For example, a configuration with k=500 and t=0.8 was found to perform closely to k=30. However, when the emphasis is on quality over diversity, nucleus sampling has been shown to outperform other decoding methods, as recommended in [99]. Based on previous findings, this study investigated the efficacy of eight decoding methods (refer to Table 3), encompassing temperature sampling, top-k sampling, and nucleus sampling, in the generation of intervention messages for smoking cessation. Specifically, in version 3, we adopted the top-p sampling value recommended by [33] and reduced the value from 0.95 (as in version 3) to 0.9 (in version 2) to examine potential improvements in model performance. Version 5 employed a temperature setting of 0.7, as suggested by [80] for message generation tasks, and we increased the value to 0.9 in version 4 to explore the impact of a temperature variation on model performance. For top-k sampling, we followed [73]’s advice, using k=40 in version 6 and adjusting it to k=30 in version 7 to study its effects. Furthermore, we adopted a hybrid approach to versions 1 and 8: version 8 was a combination of versions 5 and 6; and version 1 adjusted values of p, k, and temperature, aiming to achieve a balance between restriction and flexibility to ensure both diversity and coherency for message output.

### 3.2 Method

We replicated the message generation and evaluation process from Study 1. The recommended prompt version 1 from Study 1 was adopted. Eight decoding methods were applied to each LLM, as detailed in Table 3. In alignment with Study 1, each LLM produced messages across 100 iterations. This initial phase resulted in a total of 18,731 messages, which subsequently underwent the same filtering process (see Figure 2). The refined LLM-generated messages (N=15,575) were amalgamated with the original human-authored messages (N=899) to constitute the final evaluation sample (N=16,474). Furthermore, eight decoding methods were systematically assessed on the same dimensions in Study 1 including diversity, quality, and efficiency.

### 3.3 Results

We replicated the analysis process from Study 1, including descriptive analysis on message filtering and multiple comparisons of linguistic features and perplexity. In the initial message generation phase, each of the eight decoding methods yielded an average of 3.4 to 7.1 messages per iteration. The initial repetition filtering step discarded between 4.3% and 23.8% of these messages. Subsequent criteria-based filtering removed an additional 1.4% to 4.6% of these messages. As a result, the proportion of messages retained, relative to the initial count generated by each prompt version, ranged from 72.3% to 93.8%.

**Figure 4:**
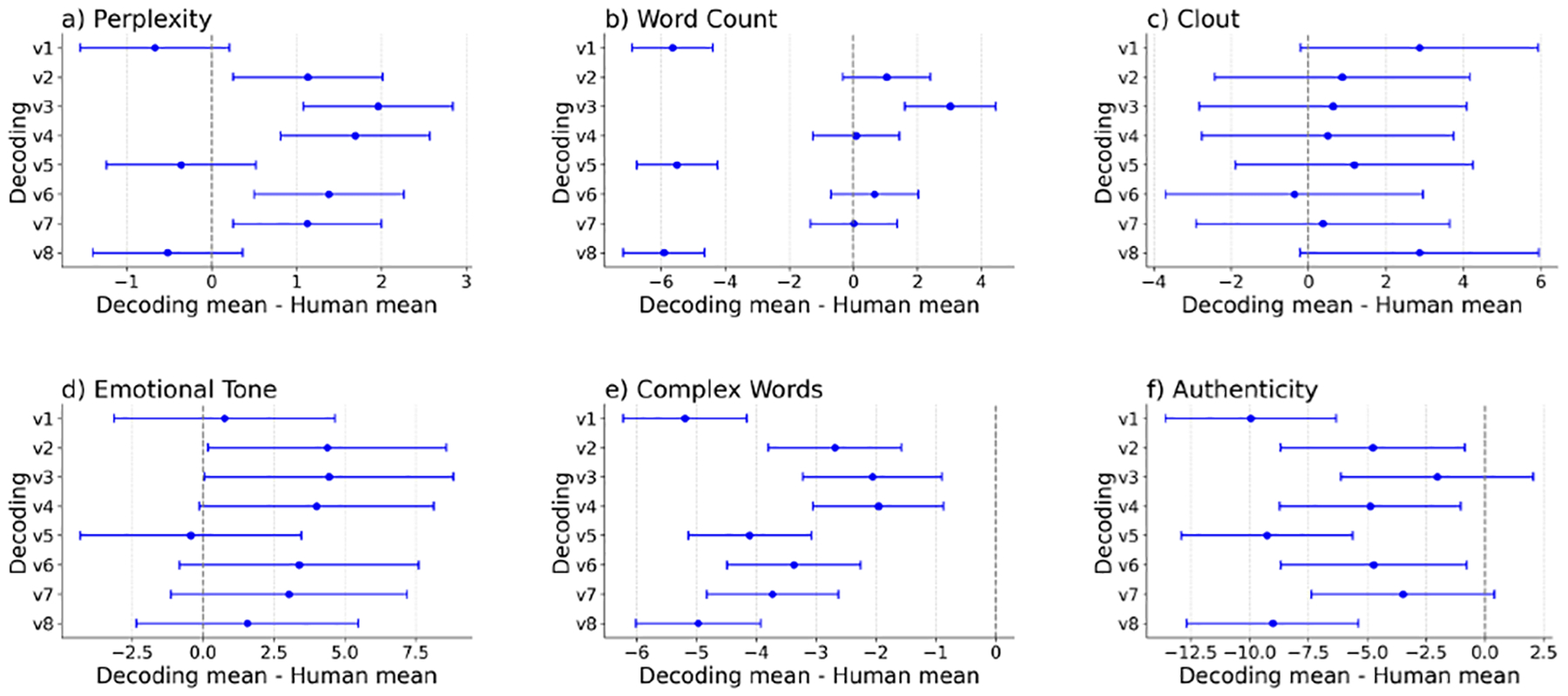
Confidence intervals for Dunnett test for decoding mean – human mean.

Five one-way analysis of variance (ANOVA) tests with post-hoc multiple comparison analyses were conducted comparing linguistic features of messages generated with 8 different decoding methods with human expert writing. Results indicated that messages generated with 8 decoding methods significantly differed from expert-written messages in terms of word count (F(8, 16148) = 137.61, p < .001, partial *η*^2^ = .06), clout (F(8, 16148) = 2.84, p < .01, partial *η*^2^ = .00), authenticity (F(8, 16148) = 14.82, p < .001, partial *η*^2^ = .01), emotional tone (F(8, 16148) = 4.18, p < .001, partial *η*^2^ = .00), and the use of complex words (F(8, 16148) = 37.95, p < .001, partial *η*^2^ = .02). Subsequent comparisons using Dunnett’s test were conducted between each decoding strategy and the expert-writing condition (Figure 4). Results indicated that, compared to expert-written messages, messages generated with decoding methods 1, 5, and 8 contained significantly fewer words (p values <.001), and messages generated with decoding method 3 had higher word count (p values <.001). Meanwhile, decoding methods 2, 4, 6, and 7 yielded messages of lengths comparable to those written by human experts. All messages generated by LLMs, regardless of the decoding strategy employed, exhibited a level of clout similar to that of expert-written messages. Messages generated using decoding methods 3 and 7 aligned with human writing in terms of authenticity, whereas messages generated with decoding methods 1, 2, 4, 5, 6, and 8 appeared more distanced and impersonal in style (p values <.05). Messages generated with decoding methods 1, 3, 4, 5, 6, 7, and 8 used neutral emotional tones like human writing, and decoding methods 2 and 3 produced more positive messages (p values <.05). All LLM-created messages used fewer complex words than human expert writing (all p values <.001). Further, the ANOVA test with post-hoc multiple comparison analysis revealed significant differences in perplexity between each decoding strategy with the expert-writing condition (F(8, 155) = 40.92, p < .001, partial *η*^2^ = .68). Post-hoc Dunnett’s test further indicated that LLMs using decoding versions 1, 5, and 8 had similar perplexity (M 5.87 to 6.18; SD .54 to .61) to human experts writing (M=6.54, SD=.63), whereas LLMs using decoding versions 2, 3, 4, 6, and 7 exhibited significantly higher perplexity (M ~ 7.66 to 8.50; SD ~ .70 to .87, all p values <.01).

### 3.4 Discussion

Results revealed that temperature sampling (version 5, t=0.7) and a hybrid approach (version 1, p=0.9, k=50, t=0.8; version 8, k=40, t=0.7) exhibited perplexity scores closely to that of the expert-writing condition. Consistent with prior studies [28, 33, 73, 80], decoding methods with higher p values (version 2, p=0.9; version 3, p=0.95) or higher temperature settings (version 4, t=0.9) were found to sample too many unlikely tokens, resulting in messages that were diverse yet incoherent. The perplexity findings also echoed the repetition filtering process: decoding methods (version 1, 5, 8) that exhibited lower and approximating-reference perplexity scores had a higher proportion of messages (7.2% to 7.7%) discarded due to repetition; conversely, decoding methods with higher perplexity scores (version 2, 3, 4) demonstrated lower rates of repetition (3.7% to 4.5%). These coherent findings between perplexity and repetition filtering reconfirm the coherence-diversity trade-off that decoding methods favoring the most probable tokens tend to produce more deterministic and less diverse message outputs, thereby raising the likelihood of repetition issues [32, 34].

Moreover, decoding versions 6 (k=40) and 7 (k=30), which were designed to produce more deterministic and coherent outputs through lower k-values, still displayed significantly higher perplexity scores compared to the expert-writing condition. On the other hand, prompt versions 1 and 8, despite employing equal or higher k-values (version 1, p=0.9, k=50, t=0.8; version 8, k=40, t=0.7), achieved perplexity scores closely approximating human performance. This suggests that a hybrid approach, incorporating nucleus sampling with top-k and temperature adjustments (as in version 1), or top-k sampling with temperature adjustments (as in version 8), may offer a more balanced model performance in terms of message quality and diversity [34].

## 4 STUDY III: EXPERT REVIEW

### 4.1 Related Work

The development of LLMs has revolutionized the NLP field and offered new opportunities for augmenting clinical practices [2, 41, 50]. However, their application in clinical contexts has raised ethical concerns related to misinformation and message quality [49]. Consequently, the evaluation of LLMs’ applicability in clinical practices necessitates a thorough examination of their safety and quality, considering subdimensions such as credibility/trustworthiness, contextual relevance, and accuracy [74]. While automated metrics like perplexity and BLEU scores are commonly used due to their cost-effectiveness, speed, and repeatability, they have been criticized for their limitations in assessing linguistic properties and overall text quality [4, 75, 76, 81]. Automated metrics can be under-informative and may not provide an accurate representation of text quality. For instance, low BLEU scores, often interpreted as indicative of poor text quality, may actually result from correct but unconventional phrasing [4]. Moreover, incremental improvements in BLEU scores, typically in the range of 1–2 points as observed in most experimental studies, corresponded to true improvements only about half of the time when subjected to human evaluations [58]. In addition, previous studies noted a lack of correlation between automated metrics and human evaluations, highlighting the limitations of relying solely on automated metrics for comprehensive LLM evaluation [55, 64, 76]. When applying LLMs in healthcare, automatic metrics fall short in assessing model performance for safety and quality concerns. Therefore, human evaluation continues to be a critical element in the assessment of LLMs, frequently serving as the gold standard against which automated metrics are compared [48, 64, 66]. This underscores the importance of incorporating human judgment into the evaluation framework to capture the nuanced aspects of text quality and safety that automated metrics may not fully encapsulate.

#### 4.1.1 Human Evaluations of Message Generation with LLMs.

Human evaluation typically involves recruiting annotators to manually assess the quality of text generated by LLMs. This approach closely approximates real-world application scenarios and provides a more comprehensive and nuanced understanding of a model’s performance. Evaluation criteria often include fluency, informativeness, relevance, coherence, accuracy, clarity, grammaticality, and appropriateness [22, 47, 56]

However, due to budgetary constraints, human evaluations often rely on crowdsourced workers from platforms such as Amazon Mechanical Turk rather than on specialized experts [23, 38, 48, 56, 95]. Although this approach is cost-effective, it has received criticisms for potentially compromising the validity of evaluations. Research indicates that non-expert evaluations may not consistently align well with expert assessments, especially for tasks that require specialized expertise, such as disinformation detection [9]. Crowdsourced annotators have been observed to focus on superficial textual features, such as text length and grammatical precision, over more substantive criteria like content accuracy and consistency [23, 95]. Further analysis has suggested that heuristics employed by non-expert annotators, including features like nonsensical and repetitive text, grammatical issues, rare bigrams, and long words, can be flawed and misleading in evaluation of LLM-generated messages [38]. This raises significant concerns in contexts that are sensitive to safety and reliability, such as messages that provide health behavior recommendations, where the deployment of LLMs necessitates rigorous evaluation to ensure both reliability and safety. The lack of specialized expertise in the evaluation process could compromise its validity, thereby posing the risk of disseminating inaccurate or unsafe information through LLM-generated outputs. Therefore, while human evaluation remains a critical component of LLM assessment, the qualifications and expertise of the evaluators should be carefully considered, especially in high-stakes contexts.

#### 4.1.2 ChatGPT.

During this study, ChatGPT, a state-of-the-art LLM, attracted considerable attention for its exceptional performance in natural language tasks [65]. Operating on a closed-source paradigm, ChatGPT is powered by GPT-3.5, an LLM trained on OpenAI’s 175-billion parameter foundation model. The training process utilized a vast corpus of text data from the internet and employed a combination of reinforcement and supervised learning methods. ChatGPT has outperformed its GPT-based predecessors in linguistic capabilities and has been evaluated for its applicability in various sectors, including healthcare education, research, and practice [44, 77]. For example, a study by Gilson et al. [29] assessed the efficacy of ChatGPT in a medical setting and found that ChatGPT was capable of utilizing logical reasoning and external information to provide accurate, coherent, and contextually relevant answers in question-answering scenarios, demonstrating a level of competency comparable to that of third-year medical students. Given ChatGPT’s proven utility in medical contexts, this study aims to further include ChatGPT as an additional LLM and examine its feasibility in generating intervention messages for smoking cessation.

### 4.2 Method

Distinct from other LLMs, ChatGPT incorporates an additional fine-tuning process utilizing reinforcement learning from human feedback [66]. This methodology involves ranking a broad spectrum of responses to diverse prompts based on human-labeled feedback, thereby enabling the model to discern between high-quality and suboptimal outputs. Given this unique fine-tuning process, it is not advisable to assume that the optimal prompt identified in Study 1, which was empirically validated with other LLMs, would yield similar results when applied to ChatGPT. To address this issue, a separate evaluation was conducted to identify the most effective prompt for ChatGPT.

In this evaluation, five prompts from Study 1 were initially tested on ChatGPT each across 20 iterations, to compare their effectiveness in message generation. The initial generation phase returned an average of 3 to 11 messages per iteration, except for prompt version 5, which returned only 1 message per iteration. Repetition filtering removed 17% of the messages generated using prompt version 1, but had no impact on messages generated with other prompt versions. Criteria-based filtering further discarded 1% of the messages generated with prompt version 2. Prompt versions 3 and 4 demonstrated superior performance, yielding multiple messages per iteration, all of which passed both repetition and criteria-based filtering, thereby achieving a 100% pass rate. Compared with version 3, version 4 returned more messages per iteration, thereby was recommended and applied to ChatGPT for subsequent message generation, taking into account both quality and efficacy considerations.

Following the message generation process in Study 1, prompt version 4 combined with four message exemplars randomly selected from the expert-written message library were used as input for the ChatGPT interface for 100 iterations, using its default decoding method. The initial process yielded a total of 493 messages. Subsequent message filtering discarded 0 messages for repetition (BLEU-4 ≥ 0.5) and 4 messages for context-specific criteria, resulting in a final count of 489 messages.

Founded in 2008, the Council for Tobacco Treatment Training Programs (www.ctttp.org) has developed an interdisciplinary approach to implementing training standards [84] for Tobacco Treatment Specialists (TTS). The TTS training ensures that TTS are well-prepared to address the dynamic and complex needs of tobacco users in diverse settings. Seven certified tobacco treatment specialists (TTS) were invited to participate in an expert review of messages generated by LLMs in Study 3, using expert-written messages as the benchmark (See Table 4 for examples of intervention messages and scores from expert evaluation). Messages generated using prompt version 1 and decoding version 1 from Study 2 were chosen for review due to their superior performance and efficiency. Each specialist was randomly assigned to review a set of 100 messages, with approximately 16 messages stratified and randomly selected from each of the five LLM message banks (N=624 for GPT-J-6B, N=300 for Bloom 7B1, N=498 for OPT 6.7B, N=537 for OPT 13B, and N=599 for OPT 30B), ChatGPT message bank (N=489), and the expert-written message library (N=899).

Drawing on the widely validated message evaluation protocol for health interventions [20, 42], each message was evaluated using 10-point scales for quality, accuracy, credibility, and persuasiveness. Message quality was assessed using a single item: “Considering both content and style, how well-written is the message?” with scores ranging from 1 (poorly written) to 10 (very well-written) adopted from [19]. Accuracy was assessed by a single item: “How accurate is this message? (Accurate refers to no misinformation and no factual errors)” [39]. Credibility was assessed by a single item asking “how credible does this message seem to you?” [5]. Persuasiveness was assessed by a single item asking “To what extent do you feel this message can help smokers avoid smoking? [7]. Additionally, a binary measure was employed asking whether the message met the standards in TTS training, making it ready to use in smoking cessation interventions.

### 4.3 Results

Four one-way analysis of variance (ANOVA) tests with post-hoc multiple comparison analyses were conducted to compare the message quality, accuracy, credibility, and persuasiveness of LLM-generated messages with expert-written messages. Results revealed significant differences between the LLM-generated and expert-written messages in terms of message quality (F(6, 693) = 12.93, p < .001, partial *η*^2^ = .10), accuracy (F(6, 693) = 12.75, p < .001, partial *η*^2^ = .10), credibility (F(6, 693) = 13.62, p < .001, partial *η*^2^ = .11), and persuasiveness (F(6, 693) = 13.36, p < .001, partial *η*^2^ = .10). Subsequent Dunnett’s test were conducted to compare each LLM with the expert-writing condition. Results indicated that, messages generated by ChatGPT achieved the highest mean scores across all four evaluation metrics. Specifically, ChatGPT significantly outperformed human experts in message quality (M=7.46, SD =2.22 vs. M=6.47, SD=2.59, p = .04) and demonstrated comparable performance in credibility, accuracy, and persuasiveness. Additionally, OPT-13B and OPT-30B exhibited equal performance to human experts across four evaluation metrics. Conversely, BLOOM-7B1, GPT-J-6B, and OPT-6.7B constantly underperformed human experts on four evaluations criteria. Furthermore, a Chi-Square test of independence was conducted to examine the pass rates across different LLMs in comparison to the expert-writing condition. The results indicated significant differences in pass rates, *χ*^2^(1, N=700) = 10.98, p < .001. Post-hoc analyses with Bonferroni adjustments revealed that ChatGPT achieved a significantly higher pass rate (71%) compared to the expert-writing condition (48%). OPT-30B and OPT-13B exhibited similar pass rates, with 40% for OPT-30B and 33% for OPT-13B. In contrast, OPT-6.7B, GPT-J-6B, and BLOOM-7B1 demonstrated significantly lower pass rates, registering at 25%, 22%, and 19%, respectively.

### 4.4 Discussion

Our findings offer compelling evidence for the capabilities of larger LLMs like ChatGPT, OPT-13B, and OPT-30B in generating high-quality intervention messages for smoking cessation. Notably, ChatGPT not only met but exceeded human performance in critical evaluation metrics. Specifically, it outperformed human experts in terms of message quality and generated a significantly higher rate of messages that met the TTS standards, as assessed by certified TTS. These results demonstrate the efficacy of LLMs in generating intervention messages for clinical practices. The superior performance of ChatGPT in generating ready-to-use, high-quality intervention messages implies its potential as a valuable tool for healthcare professionals in the field of tobacco treatment and suggests possibilities for broader adoption in various healthcare contexts.

## 5 GENERAL DISCUSSION AND LIMITATION

Collectively, these three studies present a thorough exploration of message generation and evaluation with LLMs in the specialized context of smoking cessation interventions. Based upon a comprehensive evaluation framework that integrates automated metrics, linguistic attributes, and expert assessments, we have demonstrated that succinct prompt with general instructions (Study 1) and a hybrid decoding approach, which incorporates nucleus sampling with top-k and temperature adjustments (Study 2), achieved optimal performance on message diversity, quality, and generation efficiency, closely approaching the level of human expert writing. Furthermore, expert review (Study 3) revealed that larger LLMs, including ChatGPT, OPT-13B and OPT-30B, can effectively emulate human expert writing to generate well-written, accurate, credible, and persuasive messages, with about half of the generated messages meeting the clinical standards to be directly adopted in smoking cessation intervention.

This research makes several substantial contributions to the field: 1) It identifies optimal prompt and decoding strategies for message generation in specialized healthcare contexts, offering generalizable insights across five state-of-art LLMs, and thereby establishing a roadmap for their effective deployment in healthcare; 2) It adopts the computational linguistic approach and introduces a comprehensive evaluation framework that assesses LLM performance in terms of message diversity, quality, and efficiency, offering a robust framework for LLM evaluations for future studies; 3) It underscores the potential of LLMs to augment health interventions by generating a substantial volume of high-quality, accurate, and persuasive messages, thereby offer the opportunity to enhance the scalability and efficacy of future health interventions; and 4) It offers practical guidelines for healthcare professionals on the adoption of LLMs in clinical practice, addressing key considerations such as model optimization, message filtering, comprehensive evaluation, and expert review. Importantly, our findings demonstrate that advanced LLMs like ChatGPT, OPT-13B, and OPT-30B can serve as valuable adjuncts in clinical settings for content generation. Moreover, this research addresses, for the first time, the safety and quality concerns associated with the use of LLM-generated messages in healthcare contexts through the rigorous review from clinicians.

### 5.1 Optimizing LLMs for Message Generation in Healthcare

Optimizing LLMs for message generation necessitates a nuanced understanding of both the nature of the messages to be generated and the specific characteristics of the LLMs employed. Results from Study 1 indicate that a succinct prompt, featuring general instructions placed prior to message exemplars, yielded optimal performance in the generation of healthcare-related messages. This observation is consistent with existing literature [45, 60, 61, 71, 91], which posits that, in the context of few-shot learning, LLMs derive greater benefit from message exemplars for learning the distribution of input text, as opposed to relying heavily on explicit task instructions. This finding is particular true for the generation of specialized content, such as healthcare messages, which exhibit unique linguistic patterns that distinguish them from general, everyday discourse [68, 87, 88]. In a similar vein, we hypothesize that the suboptimal performance observed with prompt version 4, which positioned the task instruction after the message exemplars, may be due to a disruption of in-context learning of the latent concept about smoking cessation. A Bayesian interpretation of the in-context learning posits that a list of independent and identically distributed (IDD) training examples provided in the prompt enables the LLM to locate a hidden concept shared among these examples [94, 96]. The task instruction after these examples may have shifted the posterior probability distribution of the learned concept, leading to inferior performance.

Intriguingly, despite its suboptimal performance across five other LLMs, prompt version 4 yielded the most effective results when applied to ChatGPT. This underscores the notion that prompt selection should be tailored to the specific attributes of the LLM in use. ChatGPT’s unique fine-tuning process [66], which enables it to better interpret and act upon task instructions within the prompt. In our task, the instruction after the examples may have allowed ChatGPT to better act on the latent concept learned from the IID training examples. This capability distinguishes ChatGPT from other LLMs and aligns with recent findings in the literature [91].

Moreover, Study 2 identified the optimal decoding method for generating intervention messages, specifically recommending a hybrid decoding approach that combines nucleus sampling with top-k and temperature adjustments (decoding version 1, p=0.9, k=50, t=0.8). This approach outperformed the restricted top-k sampling methods (decoding versions 6, k=40 and version 7, k=30) by offering a balanced performance in terms of both message quality and diversity, consistent with existing literature [34]. Given the critical importance of safety and quality in healthcare messaging, our study aimed for LLMs to closely emulate human expert writing. The decoding method recommended in Study 2, tailored to this specific requirement, utilizes a restrictive strategy to prioritize accuracy over creativity. Consistent with prior research, our study suggests that different tasks necessitate different decoding methods. For instance, in tasks where accuracy is paramount, such as summarization, translation, or message generation for a healthcare context, more restrictive decoding methods are often employed (e.g., k=2, as in [28]). Conversely, tasks prioritizing diversity, like creative writing, advertising copywriting, or storytelling, may benefit from more flexible decoding methods (e.g., k=100, as cited in [10]). Therefore, we suggest customizing decoding methods to align with the specific requirements of the NLP task in future studies.

### 5.2 Evaluating LLMs in Healthcare Contexts

The evaluation of LLMs is important for ensuring their safe and effective deployment in healthcare settings. Traditional automatic metrics like perplexity or BLEU often prove inadequate for comprehensively assessing model performance in the context of automatic message generation [55, 64, 76]. Moreover, these metrics can yield results that are difficult to interpret [4]. To address these limitations, our study proposes the augmentation of automatic metrics with computational linguistic analysis. This multi-faceted approach allows for a more nuanced and interpretable understanding of model performance. Specifically, results indicate the incompetence of perplexity to correctly reflect subtle messaging style of linguistic features. This means that LLMs can produce messages that differ significantly from those written by experts, despite having similar perplexity. Conversely, a significant discrepancy in perplexity scores between LLMs and human experts does not necessarily indicate differences in linguistic patterns either. Our work integrates computational linguistic analysis to contextualize model performance in message generation tasks, focusing on critical linguistic features. This approach provides a more holistic and interpretable evaluation of Large Language Models’ (LLMs) capabilities.

Furthermore, it is worth noting that even in the best-performing case of ChatGPT, approximately 30% of generated messages did not meet the TTS standards for clinical use. Therefore, while LLMs can augment smoking cessation interventions, our results emphasize the importance of subjecting LLM-generated messages to systematic evaluation before presenting to patients. In particular, we propose a practical guideline for healthcare professionals on the integration of LLMs in clinical practice. We advocate that the process of LLM-based automated message generation should encompass model optimization, message filtering, comprehensive evaluation, and expert review to ensure its safety, quality, and effectiveness for clinical application.

### 5.3 Limitations

The current study is subject to several limitations. First, this study primarily focused on prompt engineering and decoding optimization in the message generation process. Subsequent research may improve the model performance through two aspects:1) incorporating model fine-tuning as a third strategy, and 2) pre-processing the human-written messages before prompting. In particular, expert-written intervention messages adopted in this study were originally designed for a smartphone-based smoking cessation intervention, which covered a wide range of topics, such as coping with smoking urge, motivation to quit smoking, mood change and anxiety during withdrawal. Therefore, to improve model performance, future studies could pre-categorize expert-written messages by topics and increase the coherency of message examples in prompting. Second, despite the engagement of certified tobacco treatment specialists for the expert assessment of messages generated by LLMs, the sample size of these specialists was relatively small (N=7, reviewing a total of 700 messages). This limitation was contingent on the availability of TTS specialists within our tobacco treatment group, which could introduce bias and potentially compromise the robustness of the evaluations. Consequently, the assessments may not comprehensively represent the spectrum of opinions and clinical practices within the field. Third, the cross-sectional nature of the expert review, combined with the volume of messages (N=100) assigned to each specialist for evaluation, resulted in an out-of-context assessment of the messages. For instance, certain messages may have been crafted to address specific scenarios, such as experiencing the social pressure to smoke or coping with stress eating during nicotine withdrawal. Consequently, the validity of the evaluation concerning message persuasiveness was compromised when devoid of this contextual information. Future research could better balance between the quantity of messages and the need for in-context review. By providing contextual information specific to each message, evaluators could assess the messages’ effectiveness within the scenarios they are intended to address. This approach would likely yield a more nuanced and accurate understanding of the messages’ persuasive effectiveness, thereby improving the validity of future research.

## 6 CONCLUSION

We conducted a systematic examination of message generation and evaluation process with LLMs to address two critical questions regarding the applicability of LLMs in healthcare: 1) How to optimize LLMs to mimic human expert writing and 2) Do LLM-generated messages meet clinical standards to be safely implemented in tobacco treatments? Through three studies on prompt engineering, decoding optimization, and expert review, we identified optimal prompt and decoding strategies across state-of-art LLMs for message generation in healthcare, using human expert writing as the benchmark. We proposed a comprehensive evaluation framework encompassing automatic metrics, linguistic attributes, and expert review to assess LLMs in healthcare context on diversity, quality, and efficiency. Further, LLM-generated messages were evaluated by certified TTS on message quality, accuracy, credibility, and persuasiveness. Drawing upon their extensive clinical experiences in tobacco treatment counseling, the TTS concluded that larger LLMs, including ChatGPT, OPT-13B and OPT-30B, can effectively emulate human expert writing to generate well-written, accurate, credible, and persuasive messages, thereby demonstrating their applicability in clinical practices.

## Figures and Tables

**Table 1:** Five version of prompts used in Study 1

	Task		
Version	Position	Instruction	Type of list
v1	start	Messages:	Numbered
v2	start	Messages to help you quit smoking:	Numbered
v3	start	Write motivational messages to encourage people to quit smoking:	Numbered
v4	end	Write messages like the previous ones:	Numbered
v5	start	Task: Write messages that are on the same topic.	Labelled

**Table 2:** Example of a prompt with four sample messages used in Study 1

**Prompt**	Messages to help you quit smoking:
**Sample messages**	Remember your progresss! How long has it been since you last smoked? Calculate the number of hours and give yourself credit for your hard work! When you feel stressed, a call to a family member or friend could be a good distraction. Even after you are smoke free for a while, you may still feel an urge to smoke. The important thing to remember is that having an urge to smoke does not mean you have to smoke! Nicotine lozenges will help you cope with urges to smoke, but you may still feel urges at times. These urges are only temporary, though. They will pass. Don’t let them spoil your hard work.

**Table 3:** Parameters for the decoding methods used in Study 2

Version	top p^1^	top k^2^	temperature^3^
v1	0.90	50	0.8
v2	0.90	NU	NU
v3	0.95	NU	NU
v4	NU	NU	0.9
v5	NU	NU	0.7
v6	NU	40	NU
v7	NU	30	NU
v8	NU	40	0.7

Notes: NU is short for Not Used,

1 meaning p=1 in top-p sampling,

2 t=1 in temperature sampling, and

3 no capping in top-k sampling

**Table 4:** Examples of Intervention Messages and Scores from Expert Evaluation in Study 3

Creator	Examples of Intervention Messages	MQ^1^	AC^2^	CR^3^	PE^4^	TTS^5^
Human experts	Nicotine doesn’t REALLY relax you. Your body is used to nicotine, and it seems more relaxed when you feed the addiction. Ex-smokers report feeling much less nervous just a few weeks after quitting!	7	8	8	7	Yes
	Ride out the desire to smoke. It WILL go away! Don’t fool yourself into thinking you can have “just one cigarette.”	2	2	2	1	No
GPT-J-6B	If you are currently in a cigarette-smoking episode, you have a few options:	10	10	10	10	Yes
	If you are not in the mood to talk to someone, just talk to yourself. Try to distract yourself with a phone call or text message. Get outside and move your body. If you are too uncomfortable to talk to yourself, talk to a friend, family member, or counselor. Call or text a friend and say you are feeling down. Let them know you are ready to quit smoking. If you are feeling too down, call your counselor.					
	Nicotine replacement therapy (such as patches, gums, and lozenges) may help reduce the amount of nicotine in your blood and help you feel less anxious during a quit attempt.	1	1	1	1	No
BLOOM-7B1	Smoking has a direct relationship to heart disease and stroke.	9	9	9	7	Yes
	Quit smoking in the morning. If you start smoking in the morning, you have more chances of quitting.	1	2	1	2	No
OPT-6.7B	If you’re going to quit smoking, try to quit by doing something fun. You’re less likely to smoke when you’re having fun!	10	8	9	8	Yes
	If you need help, you can call a quit line. If you are in a pinch, you can also call a quit line. If you have a quit partner, ask them to call a quit line and check in on you.	1	2	1	2	No
OPT-13B	If you smoke, you are not alone. There are millions of people who smoke and are trying to quit.	8	8	8	7	Yes
	The sooner you quit smoking, the better your chances of quitting for good.	3	1	1	1	No
OPT-30B	If you are feeling anxious, depressed, or irritable, you may have withdrawal symptoms. Talk to your health care provider or a counselor about how to cope with these feelings.	9	9	9	9	Yes
	Do not worry about having cravings. It is normal.	3	2	2	1	No
ChatGPT	Remember that quitting smoking is a journey, not a destination. Take it one day at a time and celebrate every small victory along the way.	10	10	10	10	Yes
	Consider the financial benefits of quitting smoking. Not only will you save money on cigarettes, but you’ll also save money on healthcare costs in the long run.	10	1	3	3	No

Notes:

1 MQ: Message Quality,

2 AC: Accuracy,

3 CR: Credibility,

4 PE: Persuasiveness,

5 TTS: Meet TTS training standards to use in smoking cessation intervention

## CCS CONCEPTS

- Human-centered computing → Empirical studies in HCI.

## References

[1] Abroms Lorien C., Lee Westmaas J, Bontemps-Jones Jeuneviette, Ramani Rathna, and Mellerson Jenelle. 2013. A Content Analysis of Popular Smartphone Apps for Smoking Cessation. American Journal of Preventive Medicine 45, 6 (2013), 732–736. 10.1016/j.amepre.2013.07.008 24237915 PMC3836190

[2] Agrawal Monica, Hegselmann Stefan, Lang Hunter, Kim Yoon, and Sontag David. [n. d.]. Large language models are few-shot clinical information extractors (Proceedings of the 2022 Conference on Empirical Methods in Natural Language Processing). Association for Computational Linguistics, 1998–2022. 10.18653/v1/2022.emnlp-main.130

[3] Ahmed Imtihan, Keilty Eric, Cooper Carolynne, Selby Peter, and Rose Jonathan. 2022. Generation and Classification of Motivational-Interviewing-Style Reflections for Smoking Behaviour Change Using Few-Shot Learning with Transformers. IEEE JOURNAL OF BIOMEDICAL AND HEALTH INFORMATICS (2022).

[4] Ananthakrishnan R, Bhattacharyya Pushpak, Sasikumar M, and Shah Ritesh M. 2007. Some issues in automatic evaluation of english-hindi mt: more blues for bleu. (2007).

[5] Appelman Alyssa and Shyam Sundar S. 2016. Measuring Message Credibility: Construction and Validation of an Exclusive Scale. Journalism & Mass Communication Quarterly 93, 1 (March 2016), 59–79. 10.1177/1077699015606057

[6] Bahl Lalit R., Jelinek Frederick, and Mercer Robert L.. 1983. A Maximum Likelihood Approach to Continuous Speech Recognition. IEEE Transactions on Pattern Analysis and Machine Intelligence PAMI-5, 2 (March 1983), 179–190. 10.1109/TPAMI.1983.4767370 21869099

[7] Baig Sabeeh A, Noar Seth M, Gottfredson Nisha C, Boynton Marcella H, Ribisl Kurt M, and Brewer Noel T. 2019. UNC Perceived Message Effectiveness: Validation of a Brief Scale. Annals of Behavioral Medicine 53, 8 (July 2019), 732–742. 10.1093/abm/kay080 30321252 PMC6636889

[8] Baumgartner Jason, Zannettou Savvas, Keegan Brian, Squire Megan, and Blackburn Jeremy. 2020. The Pushshift Reddit Dataset. Proceedings of the International AAAI Conference on Web and Social Media 14, 1 (2020), 830–839. 10.1609/icwsm.v14i1.7347

[9] Belz Anja and Reiter Ehud. [n. d.]. Comparing Automatic and Human Evaluation of NLG Systems (11th Conference of the European Chapter of the Association for Computational Linguistics). Association for Computational Linguistics, 313–320. https://aclanthology.org/E06-1040

[10] Bhandari Prabin and Brennan Hannah Marie. 2023. Trustworthiness of Children Stories Generated by Large Language Models. arXiv preprint arXiv:2308.00073 (2023).

[11] Biderman Stella, Bicheno Kieran, and Gao Leo. 2022. Datasheet for the Pile. arXiv preprint arXiv:2201.07311 (2022).

[12] Bommasani Rishi, Hudson Drew A, Adeli Ehsan, Altman Russ, Arora Simran, von Arx Sydney, Bernstein Michael S, Bohg Jeannette, Bosselut Antoine, and Brunskill Emma. 2021. On the opportunities and risks of foundation models. arXiv preprint arXiv:2108.07258 (2021).

[13] Boyd Ryan L, Ashokkumar Ashwini, Seraj Sarah, and Pennebaker James W. 2022. The development and psychometric properties of LIWC-22. Austin, TX: University of Texas at Austin (2022), 1–47.

[14] Brown Onikia N., O’Connor Lauren E., and Savaiano Dennis. 2014. Mobile MyPlate: A Pilot Study Using Text Messaging to Provide Nutrition Education and Promote Better Dietary Choices in College Students. Journal of American College Health 62, 5 (July 2014), 320–327. 10.1080/07448481.2014.899233 24654921

[15] Brown Tom B, Mann Benjamin, Ryder Nick, Subbiah Melanie, Kaplan Jared, Dhariwal Prafulla, Neelakantan Arvind, Shyam Pranav, Sastry Girish, and Askell Amanda. 2020. Language models are few-shot learners. arXiv preprint arXiv:2005.14165 (2020).

[16] Businelle Michael S, Ma Ping, Kendzor Darla E, Frank Summer G, Vidrine Damon J, and Wetter David W. 2016. An ecological momentary intervention for smoking cessation: evaluation of feasibility and effectiveness. Journal of Medical Internet Research 18, 12 (2016), e6058. 10.2196/jmir.6058 PMC5187451 27956375

[17] Businelle Michael S., Ma Ping, Kendzor Darla E., Reitzel Lorraine R., Chen Minxing, Lam Cho Y., Bernstein Ira, and Wetter David W.. 2014. Predicting Quit Attempts Among Homeless Smokers Seeking Cessation Treatment: An Ecological Momentary Assessment Study. Nicotine & Tobacco Research 16, 10 (2014), 1371–1378. 10.1093/ntr/ntu088 24893602 PMC4207873

[18] Caccia Massimo, Caccia Lucas, Fedus William, Larochelle Hugo, Pineau Joelle, and Charlin Laurent. 2020. LANGUAGE GANS FALLING SHORT. (2020).

[19] Cacioppo John T, Petty Richard E, and Morris Katherine J. 1983. Effects of need for cognition on message evaluation, recall, and persuasion. Journal of personality and social psychology 45, 4 (1983), 805.

[20] Cappella Joseph N. 2018. Perceived Message Effectiveness Meets the Requirements of a Reliable, Valid, and Efficient Measure of Persuasiveness. Journal of Communication 68, 5 (2018), 994–997. 10.1093/joc/jqy044 30479403 PMC6241506

[21] Carreiro Stephanie, Newcomb Mark, Leach Rebecca, Ostrowski Simon, Boudreaux Edwin D., and Amante Daniel. 2020. Current reporting of usability and impact of mHealth interventions for substance use disorder: A systematic review. Drug and Alcohol Dependence 215 (Oct. 2020), 108201. 10.1016/j.drugalcdep.2020.108201 32777691 PMC7502517

[22] Chang Shuo, Dai Peng, Chen Jilin, and Chi Ed H. 2015. Got many labels? Deriving topic labels from multiple sources for social media posts using crowdsourcing and ensemble learning. In Proceedings of the 24th International Conference on World Wide Web. 397–406.

[23] Clark Elizabeth, August Tal, Serrano Sofia, Haduong Nikita, Gururangan Suchin, and Smith Noah A. 2021. All that’s’ human’is not gold: Evaluating human evaluation of generated text. arXiv preprint arXiv:2107.00061 (2021).

[24] Clarkson Philip and Robinson Tony. 1999. Towards improved language model evaluation measures. In 6th European Conference on Speech Communication and Technology (Eurospeech 1999). ISCA, 1927–1930. 10.21437/Eurospeech.1999-423

[25] Clavié Benjamin, Ciceu Alexandru, Naylor Frederick, Soulié Guillaume, and Brightwell Thomas. [n. d.]. Large Language Models in the Workplace: A Case Study on Prompt Engineering for Job Type Classification. In International Conference on Applications of Natural Language to Information Systems. Springer, 3–17.

[26] Cohn Michael A., Mehl Matthias R., and Pennebaker James W.. 2004. Linguistic Markers of Psychological Change Surrounding September 11, 2001. Psychological Science 15, 10 (Oct. 2004), 687–693. 10.1111/j.0956-7976.2004.00741.x 15447640

[27] DeLucia Alexandra, Mueller Aaron, Li Xiang Lisa, and Sedoc João. [n. d.]. Decoding Methods for Neural Narrative Generation (Proceedings of the 1st Workshop on Natural Language Generation, Evaluation, and Metrics (GEM 2021)). Association for Computational Linguistics, 166–185. 10.18653/v1/2021.gem-1.16

[28] Fan Angela, Lewis Mike, and Dauphin Yann. [n. d.]. Hierarchical Neural Story Generation (Proceedings of the 56th Annual Meeting of the Association for Computational Linguistics (Volume 1: Long Papers)). Association for Computational Linguistics, 889–898. 10.18653/v1/P18-1082

[29] Gilson Aidan, Safranek Conrad W, Huang Thomas, Socrates Vimig, Chi Ling, Taylor Richard Andrew, and Chartash David. 2023. How Does ChatGPT Perform on the United States Medical Licensing Examination? The Implications of Large Language Models for Medical Education and Knowledge Assessment. JMIR Medical Education 9 (Feb. 2023), e45312. 10.2196/45312 36753318 PMC9947764

[30] Gonen Hila, Iyer Srini, Blevins Terra, Smith Noah A., and Zettlemoyer Luke. 2022. Demystifying Prompts in Language Models via Perplexity Estimation. http://arxiv.org/abs/2212.04037. arXiv:2212.04037 [cs]

[31] Graham Amanda L, Papandonatos George D, Jacobs Megan A, Amato Michael S, Cha Sarah, Cohn Amy M, Abroms Lorien C, and Whittaker Robyn. 2020. Optimizing Text Messages to Promote Engagement With Internet Smoking Cessation Treatment: Results From a Factorial Screening Experiment. Journal of Medical Internet Research 22, 4 (April 2020), e17734. 10.2196/17734 32238338 PMC7386536

[32] Hashimoto Tatsunori B., Zhang Hugh, and Liang Percy. [n. d.]. Unifying Human and Statistical Evaluation for Natural Language Generation (Proceedings of the 2019 Conference of the North American Chapter of the Association for Computational Linguistics: Human Language Technologies, Volume 1 (Long and Short Papers)). Association for Computational Linguistics, 1689–1701. 10.18653/v1/N19-1169

[33] Holtzman Ari, Buys Jan, Du Li, Forbes Maxwell, and Choi Yejin. [n. d.]. The Curious Case of Neural Text Degeneration. In International Conference on Learning Representations.

[34] Holtzman Ari, Buys Jan, Du Li, Forbes Maxwell, and Choi Yejin. 2020. The Curious Case of Neural Text Degeneration. http://arxiv.org/abs/1904.09751 arXiv:1904.09751 [cs].

[35] Hébert Emily T, Ra Chaelin K, Alexander Adam C, Helt Angela, Moisiuc Rachel, Kendzor Darla E, Vidrine Damon J, Funk-Lawler Rachel K, and Businelle Michael S. 2020. A mobile Just-in-Time adaptive intervention for smoking cessation: pilot randomized controlled trial. Journal of medical Internet research 22, 3 (2020). 10.2196/16907 PMC7091024 32149716

[36] Hébert Emily T, Stevens Elise M, Frank Summer G, Kendzor Darla E, Wetter David W, Zvolensky Michael J, Buckner Julia D, and Businelle Michael S. 2018. An ecological momentary intervention for smoking cessation: the associations of just-in-time, tailored messages with lapse risk factors. Addictive behaviors 78 (2018), 30–35. 29121530 10.1016/j.addbeh.2017.10.026 PMC5783727

[37] Ippolito Daphne, Kriz Reno, Sedoc Joao, Kustikova Maria, and Callison-Burch Chris. 2019. Comparison of Diverse Decoding Methods from Conditional Language Models. In Proceedings of the 57th Annual Meeting of the Association for Computational Linguistics. Association for Computational Linguistics, Florence, Italy, 3752–3762. 10.18653/v1/P19-1365

[38] Jakesch Maurice, Hancock Jeffrey T., and Naaman Mor. 2023. Human heuristics for AI-generated language are flawed. Proceedings of the National Academy of Sciences 120, 11 (2023), e2208839120. https://doi.org/doi:10.1073/pnas.2208839120 PMC10089155 36881628

[39] Jung Eun Hwa, Walsh-Childers Kim, and Kim Hyang-Sook. 2016. Factors influencing the perceived credibility of diet-nutrition information web sites. Computers in Human Behavior 58 (2016), 37–47.

[40] Kacewicz Ewa, Pennebaker James W., Davis Matthew, Jeon Moongee, and Graesser Arthur C.. 2014. Pronoun Use Reflects Standings in Social Hierarchies. Journal of Language and Social Psychology 33, 2 (March 2014), 125–143. 10.1177/0261927X13502654

[41] Karinshak Elise, Sunny Xun Liu Joon Sung Park, and Hancock Jeffrey T. 2023. Working With AI to Persuade: Examining a Large Language Model’s Ability to Generate Pro-Vaccination Messages. Proceedings of the ACM on Human-Computer Interaction 7, CSCW1 (2023), 1–29.

[42] Kim Minji and Cappella Joseph N. 2019. Reliable, valid and efficient evaluation of media messages: Developing a message testing protocol. Journal of Communication Management 23, 3 (2019), 179–197.

[43] Kong Grace, Ells Daniel M., Camenga Deepa R., and Krishnan-Sarin Suchitra. 2014. Text messaging-based smoking cessation intervention: A narrative review. Addictive Behaviors 39, 5 (May 2014), 907–917. 10.1016/j.addbeh.2013.11.024 24462528 PMC3980005

[44] Kung Tiffany H., Cheatham Morgan, Medenilla Arielle, Sillos Czarina, De Leon Lorie, Elepaño Camille, Madriaga Maria, Aggabao Rimel, Diaz-Candido Giezel, Maningo James, and Tseng Victor. 2023. Performance of ChatGPT on USMLE: Potential for AI-assisted medical education using large language models. PLOS Digital Health 2, 2 (Feb. 2023), e0000198. 10.1371/journal.pdig.0000198 36812645 PMC9931230

[45] Lampinen Andrew, Dasgupta Ishita, Chan Stephanie, Mathewson Kory, Tessler Mh, Creswell Antonia, James McClelland Jane Wang, and Hill Felix. [n. d.]. Can language models learn from explanations in context? (Findings of the Association for Computational Linguistics: EMNLP 2022). Association for Computational Linguistics, 537–563. 10.18653/v1/2022.findings-emnlp.38

[46] Laurençon Hugo, Saulnier Lucile, Wang Thomas, Akiki Christopher, del Moral Albert Villanova, Le Scao Teven, Von Werra Leandro, Mou Chenghao, Ponferrada Eduardo González, and Nguyen Huu. 2022. The BigScience ROOTS corpus: A 1.6TB Composite Multilingual Dataset. Advances in Neural Information Processing Systems 35 (2022), 31809–31826.

[47] Lee Kyeonmin, Lee Yun Yeong, Suh Mina, Jun Jae Kwan, Park Bomi, Kim Yeol, and Choi Kui Son. 2022. Impact of COVID-19 on cancer screening in South Korea. Scientific Reports 12, 1 (July 2022), 11380. 10.1038/s41598-022-15778-3 35790880 PMC9255521

[48] Liang Percy, Bommasani Rishi, Lee Tony, Tsipras Dimitris, Soylu Dilara, Yasunaga Michihiro, Zhang Yian, Narayanan Deepak, Wu Yuhuai, and Kumar Ananya. 2022. Holistic evaluation of language models. arXiv preprint arXiv:2211.09110 (2022).

[49] Liebrenz Michael, Schleifer Roman, Buadze Anna, Bhugra Dinesh, and Smith Alexander. 2023. Generating scholarly content with ChatGPT: ethical challenges for medical publishing. The Lancet Digital Health 5, 3 (2023), e105–e106. 36754725 10.1016/S2589-7500(23)00019-5

[50] Lim Sue and Schmälzle Ralf. 2023. Artificial intelligence for health message generation: an empirical study using a large language model (LLM) and prompt engineering. Frontiers in Communication 8 (2023), 1129082.

[51] Liu Chi-Liang, Lee Hung-yi, and Yih Wen-tau. 2022. Structured prompt tuning. arXiv preprint arXiv:2205.12309 (2022).

[52] Liu Pengfei, Yuan Weizhe, Fu Jinlan, Jiang Zhengbao, Hayashi Hiroaki, and Neubig Graham. 2021. Pre-train, prompt, and predict: A systematic survey of prompting methods in natural language processing. arXiv preprint arXiv:2107.13586 (2021).

[53] Liu Yinhan, Ott Myle, Goyal Naman, Du Jingfei, Joshi Mandar, Chen Danqi, Levy Omer, Lewis Mike, Zettlemoyer Luke, and Stoyanov Veselin. 2019. RoBERTa: A robustly optimized BERT pretraining approach. arXiv preprint arXiv:1907.11692 (2019).

[54] Logan Robert IV, Balazevic Ivana, Wallace Eric, Petroni Fabio, Singh Sameer, and Riedel Sebastian. [n. d.]. Cutting Down on Prompts and Parameters: Simple Few-Shot Learning with Language Models (Findings of the Association for Computational Linguistics: ACL 2022). Association for Computational Linguistics, 2824–2835. 10.18653/v1/2022.findings-acl.222

[55] Ma Qingsong, Wei Johnny, Bojar Ondřej, and Graham Yvette. [n. d.]. Results of the WMT19 Metrics Shared Task: Segment-Level and Strong MT Systems Pose Big Challenges (Proceedings of the Fourth Conference on Machine Translation (Volume 2: Shared Task Papers, Day 1)). Association for Computational Linguistics, 62–90. 10.18653/v1/W19-5302

[56] Marvin Rebecca and Linzen Tal. [n. d.]. Targeted Syntactic Evaluation of Language Models (Proceedings of the 2018 Conference on Empirical Methods in Natural Language Processing). Association for Computational Linguistics, 1192–1202. 10.18653/v1/D18-1151

[57] Mason Michael J., Campbell Leah, Way Thomas, Keyser-Marcus Lori, Benotsch Eric, Mennis Jeremy, Zhang Jing, King Laura, May James, and Stembridge Daniel R.. 2015. Development and Outcomes of A Text Messaging Tobacco Cessation Intervention with Urban Adolescents. Substance Abuse 36, 4 (Oct. 2015), 500–506. 10.1080/08897077.2014.987946 25551337

[58] Mathur Nitika, Baldwin Timothy, and Cohn Trevor. [n. d.]. Tangled up in BLEU: Reevaluating the Evaluation of Automatic Machine Translation Evaluation Metrics. In Proceedings of the 58th Annual Meeting of the Association for Computational Linguistics. 4984–4997.

[59] Meier Tabea, Boyd Ryan L., Pennebaker James W., Mehl Matthias R., Martin Mike, Wolf Markus, and Horn Andrea B. 2019. “LIWC auf Deutsch”: The Development, Psychometrics, and Introduction of DE- LIWC2015. preprint. PsyArXiv. 10.31234/osf.io/uq8zt

[60] Min Bonan, Ross Hayley, Sulem Elior, Veyseh Amir Pouran Ben, Nguyen Thien Huu, Sainz Oscar, Agirre Eneko, Heinz Ilana, and Roth Dan. 2021. Recent Advances in Natural Language Processing via Large Pre-Trained Language Models: A Survey. http://arxiv.org/abs/2111.01243 arXiv:2111.01243 [cs].

[61] Mishra Swaroop, Khashabi Daniel, Baral Chitta, Choi Yejin, and Hajishirzi Hannaneh. [n. d.]. Reframing Instructional Prompts to GPTk’s Language (Findings of the Association for Computational Linguistics: ACL 2022). Association for Computational Linguistics, 589–612. 10.18653/v1/2022.findings-acl.50

[62] Nadeem Moin, He Tianxing, Cho Kyunghyun, and Glass James. [n. d.]. A Systematic Characterization of Sampling Algorithms for Open-ended Language Generation (Proceedings of the 1st Conference of the Asia-Pacific Chapter of the Association for Computational Linguistics and the 10th International Joint Conference on Natural Language Processing). Association for Computational Linguistics, 334–346. https://aclanthology.org/2020.aacl-main.36

[63] Newman Matthew L., Pennebaker James W., Berry Diane S., and Richards Jane M.. 2003. Lying Words: Predicting Deception from Linguistic Styles. Personality and Social Psychology Bulletin 29, 5 (May 2003), 665–675. 10.1177/0146167203029005010 15272998

[64] Novikova Jekaterina, Dušek Ondřej, Curry Amanda Cercas, and Rieser Verena. [n. d.]. Why We Need New Evaluation Metrics for NLG (Proceedings of the 2017 Conference on Empirical Methods in Natural Language Processing). Association for Computational Linguistics, 2241–2252. 10.18653/v1/D17-1238

[65] OpenAI. 2023. ChatGPT.

[66] Ouyang Long, Wu Jeffrey, Jiang Xu, Almeida Diogo, Wainwright Carroll, Mishkin Pamela, Zhang Chong, Agarwal Sandhini, Slama Katarina, Ray Alex, Schulman John, Hilton Jacob, Kelton Fraser, Miller Luke, Simens Maddie, Askell Amanda, Welinder Peter, Christiano Paul F, Leike Jan, and Lowe Ryan. 2022. Training language models to follow instructions with human feedback. In Advances in Neural Information Processing Systems, Koyejo S, Mohamed S, Agarwal A, Belgrave D, Cho K, and Oh A (Eds.), Vol. 35. Curran Associates, Inc., 27730–27744. https://proceedings.neurips.cc/paper_files/paper/2022/file/b1efde53be364a73914f58805a001731-Paper-Conference.pdf

[67] Papineni Kishore, Roukos Salim, Ward Todd, and Zhu Wei-Jing. [n. d.]. BLEU: a method for automatic evaluation of machine translation. In Proceedings of the 40th annual meeting of the Association for Computational Linguistics. 311–318.

[68] Partch Megan and Dykeman Cass. 2019. A Corpus Linguistics Study of Text Message Interventions in Substance Use Disorder Treatment. (2019).

[69] Patrick Kevin, Raab Fred, Adams Marc A, Dillon Lindsay, Zabinski Marian, Rock Cheryl L, Griswold William G, and Norman Gregory J. 2009. A Text Message–Based Intervention for Weight Loss: Randomized Controlled Trial. Journal of Medical Internet Research 11, 1 (Jan. 2009), e1. 10.2196/jmir.1100 19141433 PMC2729073

[70] Perski Olga, Hébert Emily T., Naughton Felix, Hekler Eric B., Brown Jamie, and Businelle Michael S.. 2022. Technology-mediated just-in-time adaptive interventions (JITAIs) to reduce harmful substance use: a systematic review. Addiction 117, 5 (May 2022), 1220–1241. 10.1111/add.15687 34514668 PMC8918048

[71] Prasad Archiki, Hase Peter, Zhou Xiang, and Bansal Mohit. [n. d.]. GrIPS: Gradient-free, Edit-based Instruction Search for Prompting Large Language Models (Proceedings of the 17th Conference of the European Chapter of the Association for Computational Linguistics). Association for Computational Linguistics, 3845–3864. 10.18653/v1/2023.eacl-main.277

[72] Press Ofir, Smith Noah, and Lewis Mike. 2022. Train Short, Test Long: Attention with Linear Biases Enables Input Length Extrapolation. In International Conference on Learning Representations.

[73] Radford Alec, Wu Jeffrey, Child Rewon, Luan David, Amodei Dario, and Sutskever Ilya. 2019. Language models are unsupervised multitask learners. OpenAI blog 1, 8 (2019), 9.

[74] Reddy Sandeep. 2023. Evaluating large language models for use in healthcare: A framework for translational value assessment. Informatics in Medicine Unlocked 41 (2023), 101304. 10.1016/j.imu.2023.101304

[75] Reiter Ehud. 2018. A Structured Review of the Validity of BLEU. Computational Linguistics 44, 3 (2018), 393–401. 10.1162/coli_a_00322

[76] Reiter Ehud and Belz Anja. 2009. An Investigation into the Validity of Some Metrics for Automatically Evaluating Natural Language Generation Systems. Computational Linguistics 35, 4 (2009), 529–558. 10.1162/coli.2009.35.4.35405

[77] Sallam Malik. 2023. ChatGPT Utility in Healthcare Education, Research, and Practice: Systematic Review on the Promising Perspectives and Valid Concerns. Healthcare 11, 6 (March 2023), 887. 10.3390/healthcare11060887 36981544 PMC10048148

[78] Le Scao Teven, Fan Angela, Akiki Christopher, Pavlick Ellie, Ilić Suzana, Hesslow Daniel, Castagné Roman, Luccioni Alexandra Sasha, Yvon François, and Gallé Matthias. 2022. BLOOM: A 176B-Parameter Open-Access Multilingual Language Model. arXiv preprint arXiv:2211.05100 (2022).

[79] Schick Timo and Schütze Hinrich. [n. d.]. Generating Datasets with Pretrained Language Models (Proceedings of the 2021 Conference on Empirical Methods in Natural Language Processing). Association for Computational Linguistics, 6943–6951. 10.18653/v1/2021.emnlp-main.555

[80] Schmälzle Ralf and Wilcox Shelby. 2022. Harnessing artificial intelligence for health message generation: The folic acid message engine. Journal of Medical Internet Research 24, 1 (2022), e28858. 35040800 10.2196/28858 PMC8808340

[81] Scott Donia and Moore Johanna. [n. d.]. An NLG evaluation competition? eight reasons to be cautious.

[82] Lori A Scott-Sheldon J, Lantini Ryan, Jennings Ernestine G, Thind Herpreet, Rosen Rochelle K, Salmoirago-Blotcher Elena, and Bock Beth C. 2016. Text Messaging-Based Interventions for Smoking Cessation: A Systematic Review and Meta-Analysis. JMIR mHealth and uHealth 4, 2 (May 2016), e49. 10.2196/mhealth.5436 27207211 PMC4893152

[83] Sedaghat Sam. 2023. Early applications of ChatGPT in medical practice, education and research. Clinical Medicine 23, 3 (2023), 278–279. 37085182 10.7861/clinmed.2023-0078 PMC11046560

[84] Sheffer Christine E, Payne Thomas, Ostroff Jamie S, Jolicoeur Denise, Steinberg Michael, Czabafy Sharon, Foulds Jonathan, Bars Matthew, Wassum Ken, Perry Barbara, 2016. Increasing the quality and availability of evidence-based treatment for tobacco dependence through unified certification of tobacco treatment specialists. Journal of Smoking Cessation 11, 4 (2016), 229–235.

[85] Spohr Stephanie A., Nandy Rajesh, Gandhiraj Deepthi, Vemulapalli Abhilash, Anne Sruthi, and Walters Scott T.. 2015. Efficacy of SMS Text Message Interventions for Smoking Cessation: A Meta-Analysis. Journal of Substance Abuse Treatment 56 (Sept. 2015), 1–10. 10.1016/j.jsat.2015.01.011 25720333

[86] Stephens Keri K. and Rains Stephen A.. 2011. Information and Communication Technology Sequences and Message Repetition in Interpersonal Interaction. Communication Research 38, 1 (Feb. 2011), 101–122. 10.1177/0093650210362679

[87] Tausczik Yla R. and Pennebaker James W.. 2010. The Psychological Meaning of Words: LIWC and Computerized Text Analysis Methods. Journal of Language and Social Psychology 29, 1 (March 2010), 24–54. 10.1177/0261927X09351676

[88] Toma Catalina L. and D’Angelo Jonathan D.. 2015. Tell-Tale Words:Linguistic Cues Used to Infer the Expertise of Online Medical Advice. Journal of Language and Social Psychology 34, 1 (2015), 25–45. 10.1177/0261927×14554484

[89] Vaswani Ashish, Shazeer Noam, Parmar Niki, Uszkoreit Jakob, Jones Llion, Gomez Aidan N, Kaiser Ł ukasz, and Polosukhin Illia. 2017. Attention is All you Need. In Advances in Neural Information Processing Systems, Guyon I, Von Luxburg U, Bengio S, Wallach H, Fergus R, Vishwanathan S, and Garnett R (Eds.), Vol. 30. Curran Associates, Inc. https://proceedings.neurips.cc/paper_files/paper/2017/file/3f5ee243547dee91fbd053c1c4a845aa-Paper.pdf

[90] Wang Ben. 2021. Mesh-Transformer-JAX: Model-Parallel Implementation of Transformer Language Model with JAX. https://github.com/kingoflolz/mesh-transformer-jax

[91] Webson Albert and Pavlick Ellie. [n. d.]. Do Prompt-Based Models Really Understand the Meaning of Their Prompts? (Proceedings of the 2022 Conference of the North American Chapter of the Association for Computational Linguistics: Human Language Technologies). Association for Computational Linguistics, 2300–2344. 10.18653/v1/2022.naacl-main.167

[92] White Jules, Fu Quchen, Hays Sam, Sandborn Michael, Olea Carlos, Gilbert Henry, Elnashar Ashraf, Spencer-Smith Jesse, and Schmidt Douglas C. 2023. A prompt pattern catalog to enhance prompt engineering with chatgpt. arXiv preprint arXiv:2302.11382 (2023).

[93] Whittaker Robyn, McRobbie Hayden, Bullen Chris, Rodgers Anthony, and Gu Yulong. 2016. Mobile phone-based interventions for smoking cessation. Cochrane Database of Systematic Reviews (April 2016). 10.1002/14651858.CD006611.pub4 PMC6485940 27060875

[94] What Makes In-Context Learning Work. [n. d.]. Rethinking the Role of Demonstrations: What Makes In-Context Learning Work? ([n. d.]).

[95] Wu Minghao and Aji Alham Fikri. 2023. Style Over Substance: Evaluation Biases for Large Language Models. arXiv preprint arXiv:2307.03025 (2023).

[96] Xie Sang Michael, Raghunathan Aditi, Liang Percy, and Ma Tengyu. 2021. An explanation of in-context learning as implicit bayesian inference. arXiv preprint arXiv:2111.02080 (2021).

[97] Yang Min-Jeong, Sutton Steven K., Hernandez Laura M., Jones Sarah R., Wetter David W., Kumar Santosh, and Vinci Christine. 2023. A Just-In-Time Adaptive intervention (JITAI) for smoking cessation: Feasibility and acceptability findings. Addictive Behaviors 136 (Jan. 2023), 107467. 10.1016/j.addbeh.2022.107467 36037610 PMC10246550

[98] Ybarra Michele L., Holtrop Jodi Summers, Prescott Tonya L., and Strong David. 2014. Process evaluation of a mHealth program: Lessons learned from Stop My Smoking USA, a text messaging-based smoking cessation program for young adults. Patient Education and Counseling 97, 2 (Nov. 2014), 239–243. 10.1016/j.pec.2014.07.009 25103183 PMC4254346

[99] Zhang Hugh, Duckworth Daniel, Ippolito Daphne, and Neelakantan Arvind. [n. d.]. Trading Off Diversity and Quality in Natural Language Generation (Proceedings of the Workshop on Human Evaluation of NLP Systems (HumEval)). Association for Computational Linguistics, 25–33. https://aclanthology.org/2021.humeval-1.3

[100] Zhang Susan, Roller Stephen, Goyal Naman, Artetxe Mikel, Chen Moya, Chen Shuohui, Dewan Christopher, Diab Mona, Li Xian, and Lin Xi Victoria. 2022. OPT: Open pre-trained transformer language models. arXiv preprint arXiv:2205.01068 (2022).

[101] Zhao Zihao, Wallace Eric, Feng Shi, Klein Dan, and Singh Sameer. 2021. Calibrate Before Use: Improving Few-shot Performance of Language Models., 12697–12706 pages. https://proceedings.mlr.press/v139/zhao21c.html

[102] Zhou Yongchao, Muresanu Andrei Ioan, Han Ziwen, Paster Keiran, Pitis Silviu, Chan Harris, and Ba Jimmy. [n. d.]. Large Language Models are Human-Level Prompt Engineers. In The Eleventh International Conference on Learning Representations.

[103] Zhu Kaijie, Wang Jindong, Zhou Jiaheng, Wang Zichen, Chen Hao, Wang Yidong, Yang Linyi, Ye Wei, Gong Neil Zhenqiang, and Zhang Yue. 2023. PromptBench: Towards Evaluating the Robustness of Large Language Models on Adversarial Prompts. arXiv preprint arXiv:2306.04528 (2023).

[104] Zhu Yaoming, Lu Sidi, Zheng Lei, Guo Jiaxian, Zhang Weinan, Wang Jun, and Yu Yong. 2018. Texygen: A Benchmarking Platform for Text Generation Models., 1097–1100 pages. 10.1145/3209978.3210080

